# Cancer-associated fibroblasts secrete FGF5 to inhibit ferroptosis to decrease cisplatin sensitivity in nasopharyngeal carcinoma through binding to FGFR2

**DOI:** 10.1038/s41419-024-06671-0

**Published:** 2024-04-18

**Authors:** Feng Liu, Ling Tang, Huai Liu, Yanzhu Chen, Tengfei Xiao, Wangning Gu, Hongmin Yang, Hui Wang, Pan Chen

**Affiliations:** 1 Hunan Key Laboratory of Translational Radiation Oncology, Hunan Cancer Hospital and the Affiliated Cancer Hospital of Xiangya School of Medicine, Central South University, Changsha, 410013 Hunan Province P. R. China; 2 The Animal Laboratory Center, Hunan Cancer Hospital and the Affiliated Cancer Hospital of Xiangya School of Medicine, Central South University, Changsha, 410013 Hunan Province P. R. China

**Keywords:** Tumour immunology, Tumour immunology

## Abstract

Cisplatin (DDP)-based chemoradiotherapy is one of the standard treatments for nasopharyngeal carcinoma (NPC). However, the sensitivity and side effects of DDP to patients remain major obstacles for NPC treatment. This research aimed to study DDP sensitivity regulated by cancer-associated fibroblasts (CAFs) through modulating ferroptosis. We demonstrated that DDP triggered ferroptosis in NPC cells, and it inhibited tumor growth via inducing ferroptosis in xenograft model. CAFs secreted high level of FGF5, thus inhibiting DDP-induced ferroptosis in NPC cells. Mechanistically, FGF5 secreted by CAFs directly bound to FGFR2 in NPC cells, leading to the activation of Keap1/Nrf2/HO-1 signaling. Rescued experiments indicated that FGFR2 overexpression inhibited DDP-induced ferroptosis, and CAFs protected against DDP-induced ferroptosis via FGF5/FGFR2 axis in NPC cells. In vivo data further showed the protective effects of FGF5 on DDP-triggered ferroptosis in NPC xenograft model. In conclusion, CAFs inhibited ferroptosis to decrease DDP sensitivity in NPC through secreting FGF5 and activating downstream FGFR2/Nrf2 signaling. The therapeutic strategy targeting FGF5/FGFR2 axis from CAFs might augment DDP sensitivity, thus decreasing the side effects of DDP in NPC treatment.

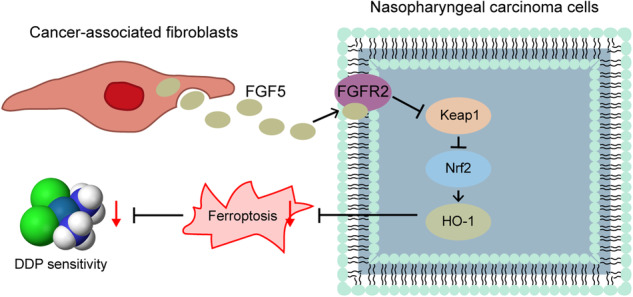

## Introduction

Nasopharyngeal carcinoma (NPC) is a frequently diagnosed head and neck cancer with high incidence in Southeast Asia and Southern China [1, 2]. Chemoradiotherapy, in particular cisplatin (DDP)-based concurrent chemoradiotherapy, is one of the standard treatments for NPC [3, 4]. Unfortunately, chemoresistance remains a major obstacle for NPC treatment [5]. Moreover, it is known that DDP-based chemotherapy has side effects to patients, such as vomiting, nausea, renal and auditory dysfunction [4]. Therefore, it is of interest to identify the combinatorial targets, thus reducing the effective dose of DDP and enhancing the DDP sensitivity in NPC.

Cancer-associated fibroblasts (CAFs), a critical component of tumor microenvironment (TME), participate in tumor growth and invasion [6]. In recent years, accumulating evidence supports that CAFs promote NPC progression [7–9]. In addition, previous studies have illustrated that CAFs contribute to DDP resistance in various cancers [10–12], however, the regulatory mechanism by which CAFs suppress DDP sensitivity in NPC remains elusive. Fibroblast growth factor (FGF) and FGF receptor (FGFR) are elevated in a variety of cancers, and associated with chemoresistance [13]. FGF/FGFR signaling pathway induces chemoresistance by facilitating angiogenesis and epithelial-mesenchymal transition (EMT), as well as suppressing apoptosis [13]. Previous study has demonstrated that CAFs result in anti-HER2 therapy resistance via FGF5/FGFR2 axis in breast cancer [14]. More importantly, FGFR2 is overexpressed in NPC tumors and cells, and its high expression is associated with unfavorable prognosis of NPC patients [15]. Lack of FGFR2 augments the effects of DDP on cell viability, cell cycle arrest and apoptosis [15]. Whether CAFs contribute to inhibit DDP sensitivity via FGF5/FGFR2 axis in NPC merits in-depth investigation.

Ferroptosis is a novel form of programmed cell death which is characterized with iron-dependent lipid peroxidation [16]. Mounting evidence has supported that DDP induces ferroptosis in different cancers [17, 18]. Recently, a study has demonstrated that DDP triggers ferroptosis in NPC cells [19], however, detailed mechanism needs further exploration. It is well-established that Keap1/Nrf2/HO-1 pathway plays a pivotal role in the suppression of ferroptosis [20], and it is also associated with the resistance to DDP [21, 22]. Nonetheless, whether Keap1/Nrf2/HO-1 signaling-mediated ferroptosis participates in regulation of DDP sensitivity in NPC remains uninvestigated.

In this study, we hypothesized that DDP promoted ferroptosis through suppressing Keap1/Nrf2/HO-1 signaling in NPC cells. CAFs-secreted FGF5 directly bound to FGFR2 in NPC cells, thus activating Keap1/Nrf2/HO-1 signaling to inhibit ferroptosis, and ultimately decreasing DDP sensitivity in NPC cells. The therapeutic strategy targeting CAFs-mediated FGF5/FGFR2 axis in TME might augment DDP sensitivity and reduce the effective dose of DDP, thus decreasing the side effects of DDP in NPC. These findings provide novel insights into CAFs and DDP sensitivity in NPC and identify potential combinatorial targets for DDP treatment.

## Results

### DDP promotes ferroptosis in NPC cells

**Fig. 1:**
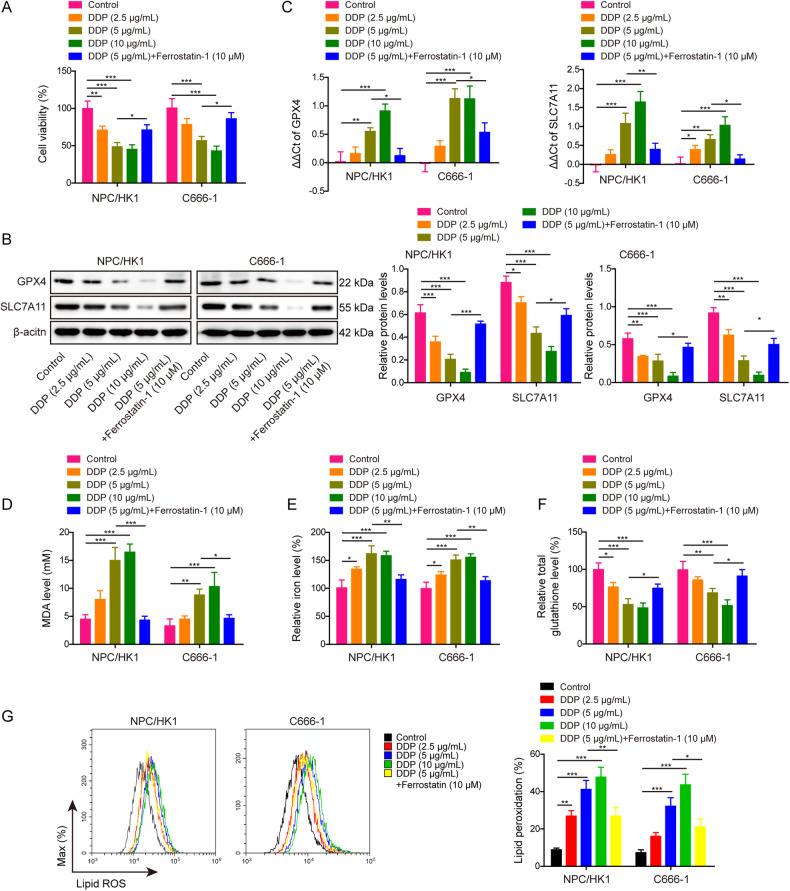
DDP promotes ferroptosis in NPC cells. NPC/HK1 and C666-1 cells were treated with 0, 2.5, 5 or 10 μg/mL DDP for 48 h, or DDP (5 μg/mL)+Ferrostatin-1 (10 μM). For the last group, NPC cells were pre-treated with 10 μM Ferrostatin-1 for 2 h, followed by the treatment of 5 μg/mL DDP for 48 h. **A** Cell viability was monitored by CCK-8 assay. **B** The protein levels of GPX4 and SLC7A11 were detected by western blot. **C** The mRNA levels of *GPX4* and *SLC7A11* were detected by qRT-PCR. **D**–**F** The levels of MDA (**D**), Fe^2+^ (**E**) and GSH (**F**) in NPC cells were measured using commercial kits. **G** NPC cells were stained with BODIPY C11 and lipid peroxidation was assessed by flow cytometry. *P < 0.05, **P < 0.01, and ***P < 0.001.

To study the effects of DDP on ferroptosis in NPC cells, two human NPC cell lines NPC/HK1 and C666-1 cells were treated with either different doses of DDP or DDP combined with ferroptosis inhibitor Ferrostatin-1 for 48 h. Cell counting kit-8 (CCK-8) assay showed that DDP reduced the viabilities of NPC cells in a dose-dependent manner, while this suppressive effect was abrogated by ferroptosis inhibitor Ferrostatin-1 (Fig. 1A). SLC7A11/GPX4 signaling is implicated in the regulation of ferroptosis [23]. Western blot and qRT-PCR revealed that DDP downregulated GPX4 and SLC7A11 protein (Fig. 1B) and mRNA (Fig. 1C) levels in NPC cells dose-dependently, whereas Ferrostatin-1 led to a rebound of GPX4 and SLC7A11 (Fig. 1B, C). Consistently, DDP increased malondialdehyde (MDA) (Fig. 1D), Fe^2+^ level (Fig. 1E) and lipid peroxidation (Fig. 1G), but decreased total glutathione (GSH) level (Fig. 1F) in NPC cells. These effects were also attenuated by Ferrostatin-1 (Fig. 1D–G). These findings indicate that DDP induces ferroptosis in NPC cells.

### DDP inhibits tumor growth via inducing ferroptosis in vivo

**Fig. 2:**
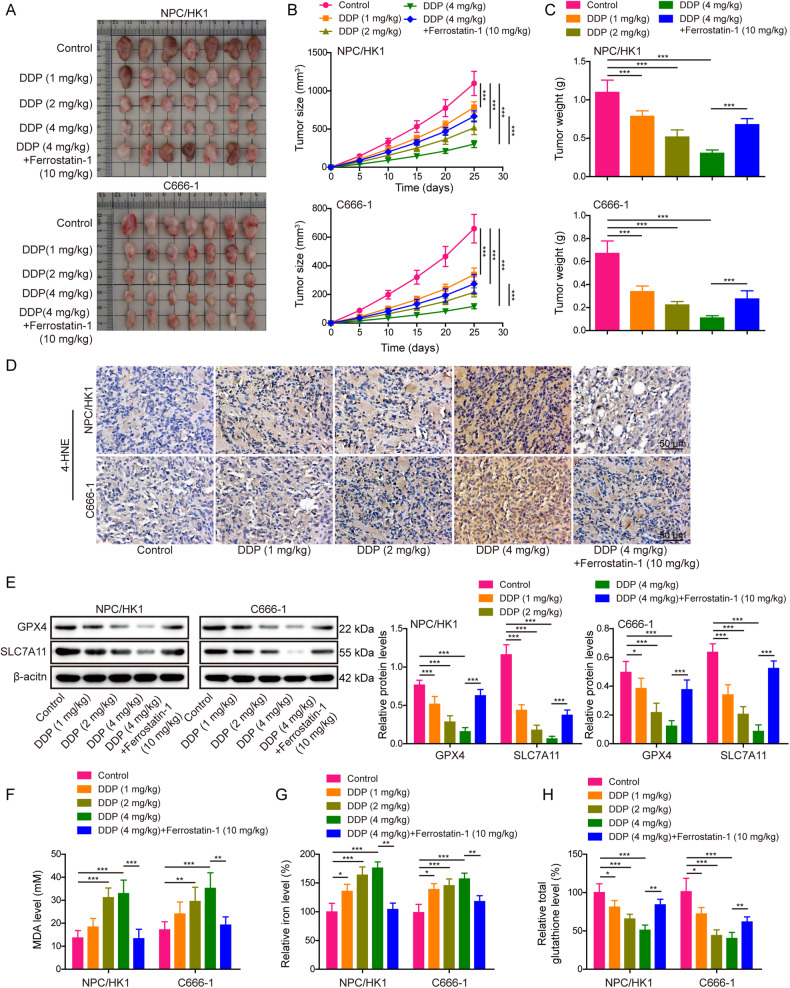
DDP inhibits tumor growth via inducing ferroptosis in vivo. Male BALB/c nude mice were randomly divided into five groups (*n* = 7 per group): control, DDP (1 mg/kg), DDP (2 mg/kg), DDP (4 mg/kg) and DDP (4 mg/kg)+Ferrostatin-1 (10 mg/kg). **A** Representative photos of xenograft tumors. **B**, **C** Quantitative analyses of tumor volume (**B**) and weight (**C**). **D** The immunoreactivity of 4-HNE in tumors was detected by IHC analysis. Scale bar: 50 μm. **E** The protein levels of GPX4 and SLC7A11 were detected by western blot. (**F**–**H**) The levels of MDA (**F**), Fe^2+^ (**G**) and GSH (**H**) in xenograft tumors were measured using commercial kits. *P < 0.05, **P < 0.01, and ***P < 0.001.

We next sought to investigate the role of DDP on tumor growth in vivo. As presented in Fig. 2A–C, DDP suppressed the growth of NPC/HK1 or C666-1 cell-derived xenograft tumor in a dose-dependent manner in which the tumor volume and weight were decreased upon DDP treatment, while this effect was partially abolished by Ferrostatin-1. Previous study has illustrated that 4-HNE is recognized as an indicator of lipid peroxidation [24]. Immunohistochemistry (IHC) analysis further showed that DDP upregulated 4-HNE level in xenograft tumors dose-dependently, whereas Ferrostatin-1 abolished DDP-induced 4-HNE expression in vivo (Fig. 2D). In line with these findings, DDP-mediated the reduction of GPX4 and SLC7A11 expression in xenograft tumors was also rescued by Ferrostatin-1 (Fig. 2E). DDP increased MDA (Fig. 2F) and Fe^2+^ (Fig. 2G) levels, but decreased GSH level (Fig. 2H) in xenograft tumors. These effects were also abrogated in the presence of Ferrostatin-1 (Fig. 2F–H). Collectively, these data suggest that DDP suppresses tumor growth via inducing ferroptosis in vivo.

### CAFs inhibit DDP-induced ferroptosis via secreting FGF5

**Fig. 3:**
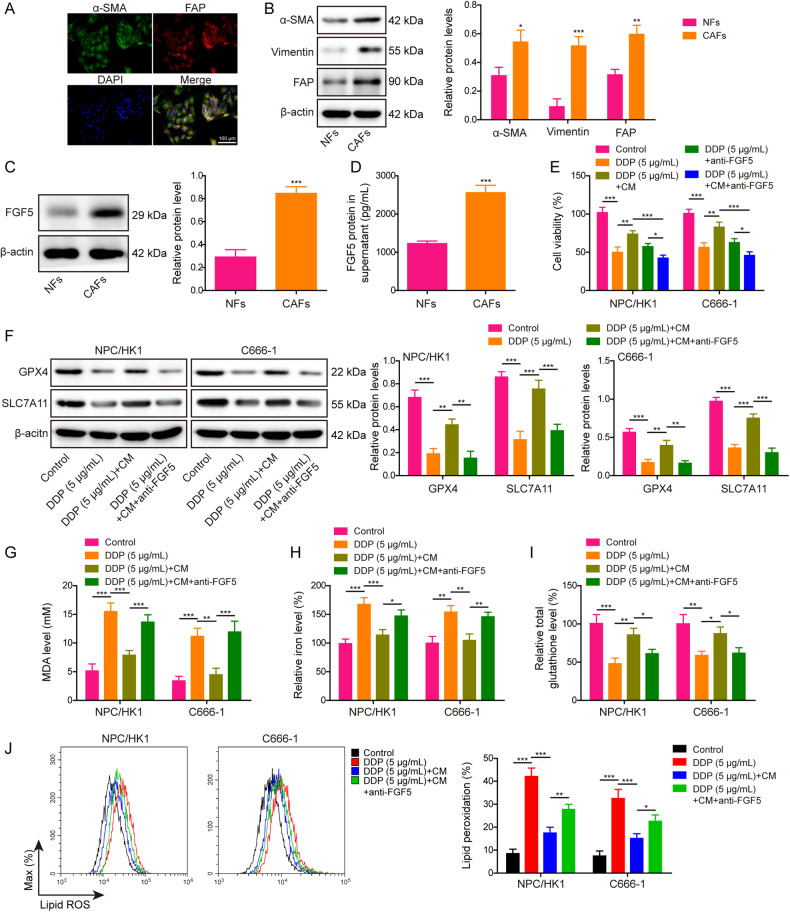
CAFs inhibit DDP-induced ferroptosis via secreting FGF5. Human CAFs and NFs were isolated from NPC tissues and the adjacent normal tissues, respectively. **A** The expression levels of α-SMA and FAP in CAFs were detected by immunofluorescence. Green, α-SMA. Red, FAP. Blue, DAPI. Scale bar: 100 μm. **B** The protein levels of α-SMA, vimentin and FAP in NFs and CAFs were detected by western blot. **C** The protein level of FGF5 in fibroblasts was detected by western blot. **D** The secretion of FGF5 in fibroblasts was assessed by ELISA assay. NPC/HK1 and C666-1 cells were divided into following groups: control, DDP (5 μg/mL), DDP (5 μg/mL)+CM and DDP (5 μg/mL)+anti-FGF5 antibody. **E** Cell viability was monitored by CCK-8 assay. **F** The protein levels of GPX4 and SLC7A11 were detected by western blot. **G**–**I** The levels of MDA (**G**), Fe^2+^ (**H**) and GSH (**I**) in NPC cells were measured using commercial kits. **J** NPC cells were stained with BODIPY C11 and lipid peroxidation was assessed by flow cytometry. *P < 0.05, **P < 0.01, and ***P < 0.001.

CAFs were isolated from NPC tissues, and immunofluorescence (Fig. 3A) showed that the fibroblast markers α-SMA and FAP were highly expressed in CAFs. In addition, western blot revealed that the protein levels of these fibroblast markers α-SMA, vimentin and FAP were much higher in CAFs, in comparison with that in normal fibroblasts (NFs) (Fig. 3B). In addition, the expression and secretion of FGF5 were remarkably upregulated in CAFs as detected by western blot (Fig. 3C) and ELISA (Fig. 3D) assays, compared with that in NFs. CCK-8 assay showed that the CAFs-derived conditioned medium (CM) reversed DDP-impaired cell viability, while this rescued effect was abrogated by FGF5 neutralizing antibody (Fig. 3E). Compared with DDP alone group, FGF5 neutralizing antibody had no significant effect cell growth (Fig. 3E). Moreover, CAFs-derived CM led to a rebound of DDP-downregulated GPX4 and SLC7A11, whereas this effect was attenuated by FGF5 neutralizing antibody (Fig. 3F). Furthermore, DDP-induced changes of MDA (Fig. 3G), Fe^2+^ (Fig. 3H), GSH (Fig. 3I) and lipid peroxidation (Fig. 3J) were reversed by CAFs-derived CM, while the protective effects of CAFs-derived CM were abolished by FGF5 neutralizing antibody (Fig. 3G–J), indicating that FGF5 in CAFs-derived CM acts as a key player in suppressing DDP-induced ferroptosis.

### CAFs-secreted FGF5 is accompanied with the activation of Keap1/Nrf2/HO-1 pathway in NPC cells

We further deciphered the mechanism by which CAFs-secreted FGF5 suppressed DDP sensitivity. Interestingly, western blot revealed that DDP upregulated Keap1 expression, and downregulated Nrf2 and HO-1 in a dose-dependent manner in NPC cells. In addition, DDP-mediated inactivation of Keap1/Nrf2/HO-1 pathway was reversed by Ferrostatin-1 (Fig. S1A). In accordance with the in vitro findings, the expression of Nrf2 in xenograft tumors was also decreased by DDP, whereas this negative effect was attenuated by Ferrostatin-1 as detected by IHC analysis (Fig. S1B). Gain- and loss-of-function experiments were next conducted to investigate the role of FGF5 in the activation of Keap1/Nrf2/HO-1 pathway. As shown in Fig. S1C, D, transfection of FGF5 overexpression or silence plasmid successfully induced or reduced FGF5 expression (Fig. S1C) and secretion (Fig. S1D) in CAFs, respectively. Among four shRNAs against FGF5 (shFGF5#1-4), shFGF5#3 with the highest knockdown efficacy was selected for the subsequent experiments (Fig. S1C, D). NPC/HK1 and C666-1 cells were then incubated with CM from indicated CAFs. As expected, CM from normal CAFs was associated with the activation of Keap1/Nrf2/HO-1 signaling pathway in which Keap1 expression was downregulated, and Nrf2 and HO-1 were upregulated, and CM derived from FGF5-overexpressing CAFs further facilitated the activation of this pathway in NPCs (Fig. S1E). By contrast, FGF5 knockdown exerted opposite effects (Fig. S1E). These findings suggest that CAFs-secreted FGF5 is accompanied with the activation of Keap1/Nrf2/HO-1 signaling pathway in NPC cells.

### CAFs-secreted FGF5 inhibits DDP-induced ferroptosis via regulating Keap1/Nrf2/HO-1 pathway in NPC cells

**Fig. 4:**
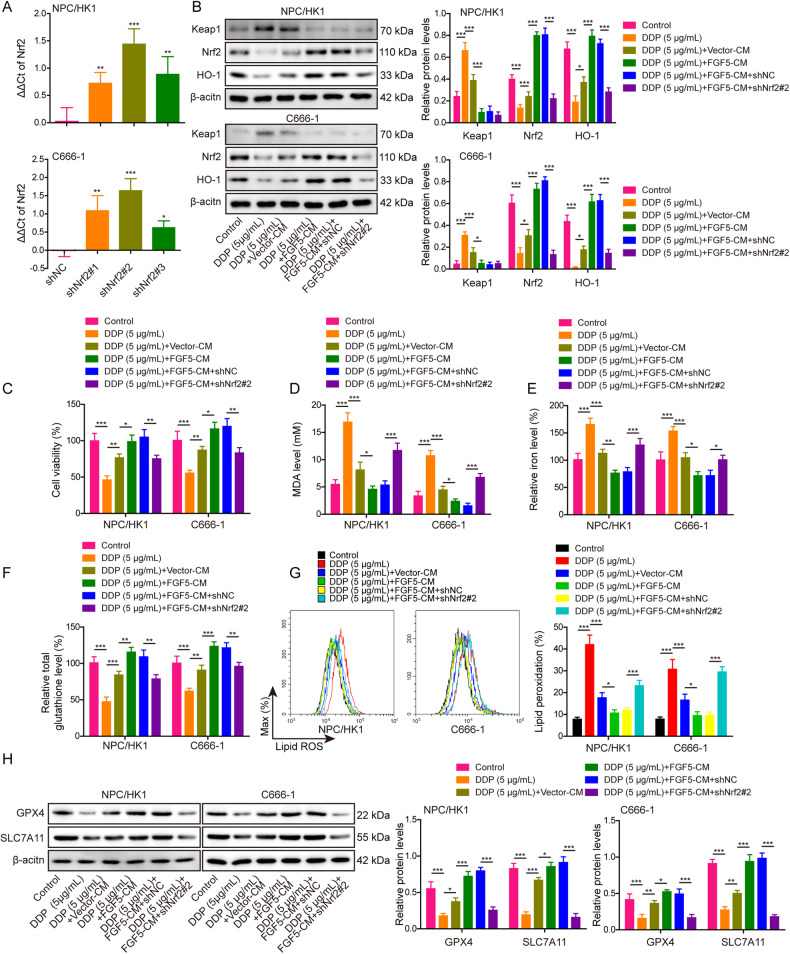
CAFs-secreted FGF5 inhibits ferroptosis caused by DDP via regulating Keap1/Nrf2/HO-1 pathway in NPC cells. **A** The mRNA level of *Nrf2* in transfected NPC cells were detected by qRT-PCR. NPC/HK1 and C666-1 cells were divided into six groups: control, DDP (5 μg/mL), DDP (5 μg/mL) + Vector-CM, DDP (5 μg/mL)+FGF5-CM, DDP (5 μg/mL)+FGF5-CM+shNC and DDP (5 μg/mL) + FGF5-CM + shNrf2#2. **B** The protein levels of Keap1, Nrf2 and HO-1 in NPC cells were detected by western blot. **C** Cell viability was monitored by CCK-8 assay. **D**–**F** The levels of MDA (**D**), Fe^2+^ (**E**) and GSH (**F**) in NPC cells were measured using commercial kits. **G** NPC cells were stained with BODIPY C11 and lipid peroxidation was assessed by flow cytometry. **H** The protein levels of GPX4 and SLC7A11 were detected by western blot. *P < 0.05, **P < 0.01, and ***P < 0.001.

We next studied the role of CAFs-secreted FGF5 in DDP-mediated inactivation of Keap1/Nrf2/HO-1 signaling. Transfection of shNrf2 reduced *Nrf2* mRNA level in NPC cells, and shNrf2#2 which is more effective in silencing Nrf2 was selected for the subsequent functional studies (Fig. 4A). As presented in Fig. 4B, DDP-inactivated Keap1/Nrf2/HO-1 signaling was reversed by CAFs-derived CM, and a more prominent effect was observed in FGF5 overexpression group. Silencing of Nrf2 counteracted FGF5-mediated activation of this pathway. Consistently, CM from FGF5-overexpressing CAFs rescued DDP-impaired cell viability (Fig. 4C), as well as DDP-mediated changes of MDA (Fig. 4D), Fe^2+^ (Fig. 4E), GSH (Fig. 4F) and lipid peroxidation (Fig. 4G), while Nrf2 knockdown reversed these protective effects (Fig. 4C–G). Western blot further showed that FGF5-overexpressing CAFs-derived CM resulted in rebounds of GPX4 and SLC7A11. As anticipated, these effects were abolished by Nrf2 knockdown (Fig. 4H). It is worth noting that the effects of FGF5 were not significantly observed in NFs. As shown in Fig. S2A, FGF5 overexpression markedly increased FGF5 expression in NFs, but the protein level of FGF5 in FGF5-overexpressing NFs was slightly lower than that in normal CAFs. The similar tendency of secreted FGF5 was observed by ELISA assay (Fig. S2B). Functional experiments showed that FGF5-overexpressing NFs-derived CM slightly counteracted the effects of DDP on cell viability (Fig. S2C), MDA (Fig. S2D), Fe^2+^ (Fig. S2E), GSH (Fig. S2F) levels and lipid peroxidation (Fig. S1G) in NPC cells, which were not significantly in most cases. The expression levels of GPX4 and SLC7A11 in NPC/HK1 and C666-1 cells were also not obviously changed by FGF5-overexpressing NFs-derived CM (Fig. S2H). Because of the concentration of FGF5 protein from NFs-derived CM quite low though overexpressing FGF5, which lower than normal CAFs, therefore this FGF5 protein concentration is not enough to suppress ferroptosis, and it is CAFs-derived FGF5, not NFs, that suppresses DDP-induced ferroptosis in NPC cells. Taken together, these data indicate that CAFs-secreted FGF5 inhibits DDP-induced ferroptosis via regulating Keap1/Nrf2/HO-1 signaling pathway in NPC cells.

### CAFs-secreted FGF5 binds to FGFR2 in NPC cells directly

**Fig. 5:**
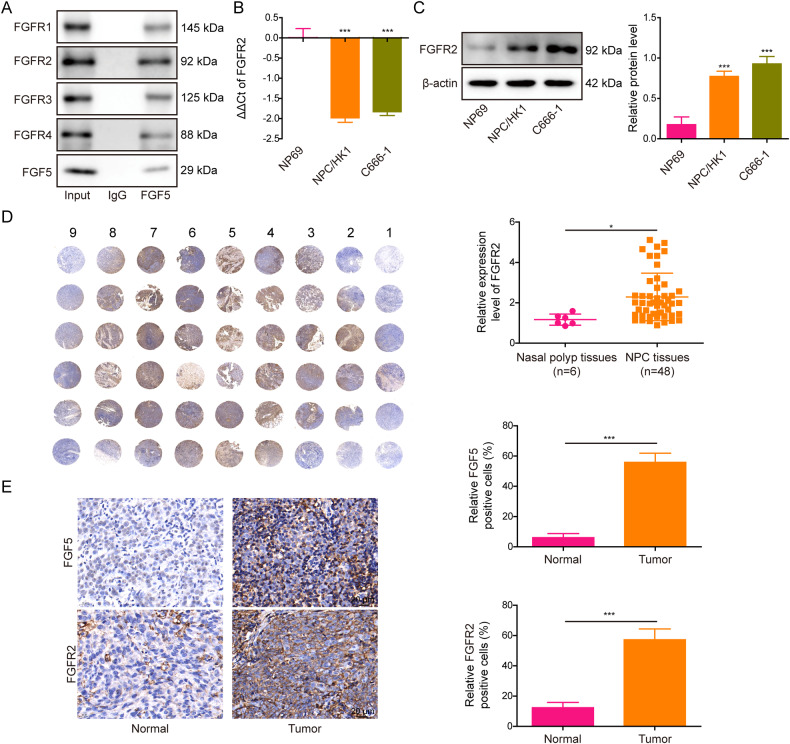
CAFs-secreted FGF5 binds to FGFR2 in NPC cells directly. **A** The interactions between FGF5 and FGFRs in NPC tissues were detected by Co-IP assay. **B** The mRNA level of *FGFR2* in N69, NPC/HK1 and C666-1 cells was detected by qRT-PCR. **C** The protein level of FGFR2 in N69, NPC/HK1 and C666-1 cells was detected by western blot. **D** The expression of FGFR2 in nasal polyp and NPC tissues was determined by tissue microarray. The first column: nasal polyp tissues. 2–9 columns: NPC tissues. **E** The immunoreactivities of FGF5 and FGFR2 in NPC tissues were detected by IHC analysis. Scale bar: 20 μm. *P < 0.05 and ***P < 0.001.

We next screened the receptors of FGF5 in NPC tissues. As shown in Fig. 5A, Co-IP showed that the tighter binding was observed between FGF5 and FGFR2 among these four receptors. FGFR2 mRNA (Fig. 5B) and protein (Fig. 5C) levels were dramatically upregulated in NPC/HK1 and C666-1 cells. Tissue microarray (TMA) analysis further confirmed the elevation of FGFR2 in NPC tissues compared with in nasal polyp tissues (the first column) (Fig. 5D). In consistent with this finding, IHC analysis further revealed the increased expression of FGF5 and FGFR2 in NPC tissues, compared with that in their normal counterparts (Fig. 5E). These data suggest that FGFR2 might be the major receptor of FGF5 in NPC.

### Overexpression of FGFR2 inhibits DDP-induced ferroptosis in NPC cells

**Fig. 6:**
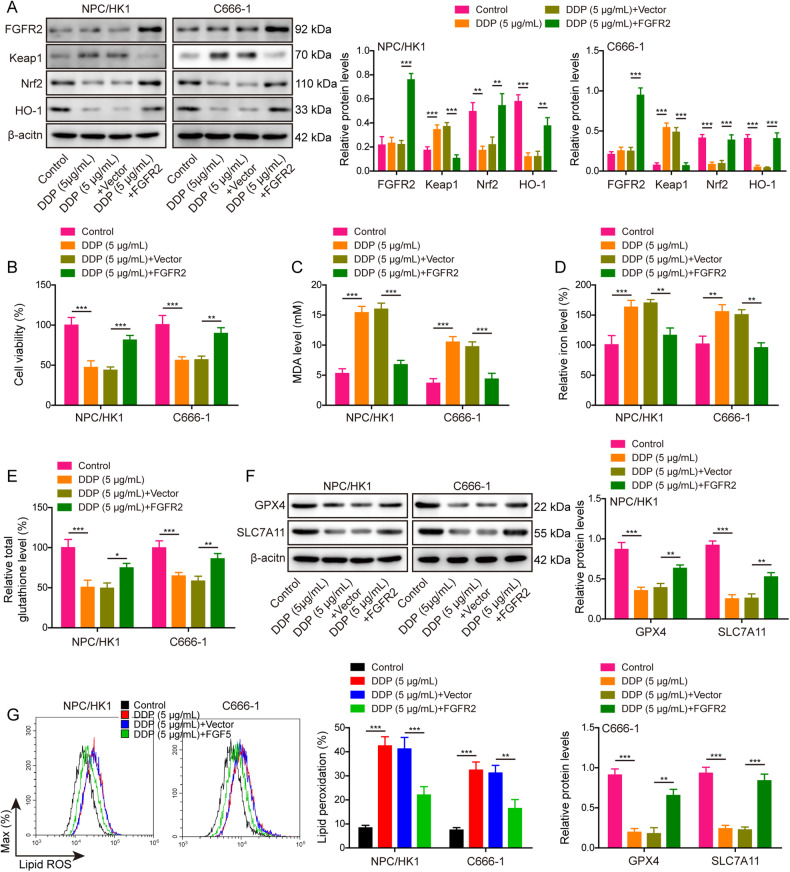
Overexpression of FGFR2 inhibits DDP-induced ferroptosis in NPC cells. NPC/HK1 and C666-1 cells were divided into four groups: control, DDP (5 μg/mL), DDP (5 μg/mL) + Vector and DDP (5 μg/mL) + FGFR2. **A** The protein levels of FGFR2, Keap1, Nrf2 and HO-1 in NPC cells were detected by western blot. **B** Cell viability was monitored by CCK-8 assay. **C**–**E** The levels of MDA (**C**), Fe^2+^ (**D**) and GSH (**E**) in NPC cells were measured using commercial kits. **F** The protein levels of GPX4 and SLC7A11 were detected by western blot. **G** NPC cells were stained with BODIPY C11 and lipid peroxidation was assessed by flow cytometry. *P < 0.05, **P < 0.01, and ***P < 0.001.

To delineate the role of FGFR2 in DDP-induced ferroptosis, overexpression of FGFR2 was conducted in NPC/HK1 and C666-1 cells upon DDP treatment. As anticipated, FGFR2 overexpression successfully increased FGFR2 protein level and it is worth noting that DDP had no remarkable effect on FGFR2 expression in NPC cells (Fig. 6A). Overexpression of FGFR2 attenuated the inhibition of Keap1/Nrf2/HO-1 pathway caused by DDP in NPC cells (Fig. 6A). CCK-8 assay revealed that DDP-impaired viabilities of NPC cells were rescued by FGFR2 overexpression (Fig. 6B). Similarly, FGFR2 overexpression protected against DDP-induced changes of MDA (Fig. 6C), Fe^2+^ (Fig. 6D) and GSH (Fig. 6E). Western blot showed that FGFR2 overexpression resulted in rebounds of GPX4 and SLC7A11 in NPC cells (Fig. 6F). Additionally, the lipid peroxidation increased by DDP was rescued by FGFR2 overexpression as detected by flow cytometry (Fig. 6G). Collectively, these findings indicate that FGFR2 overexpression suppresses DDP-induced ferroptosis in NPC cells.

### CAFs inhibit DDP-induced ferroptosis via FGF5/FGFR2 axis

**Fig. 7:**
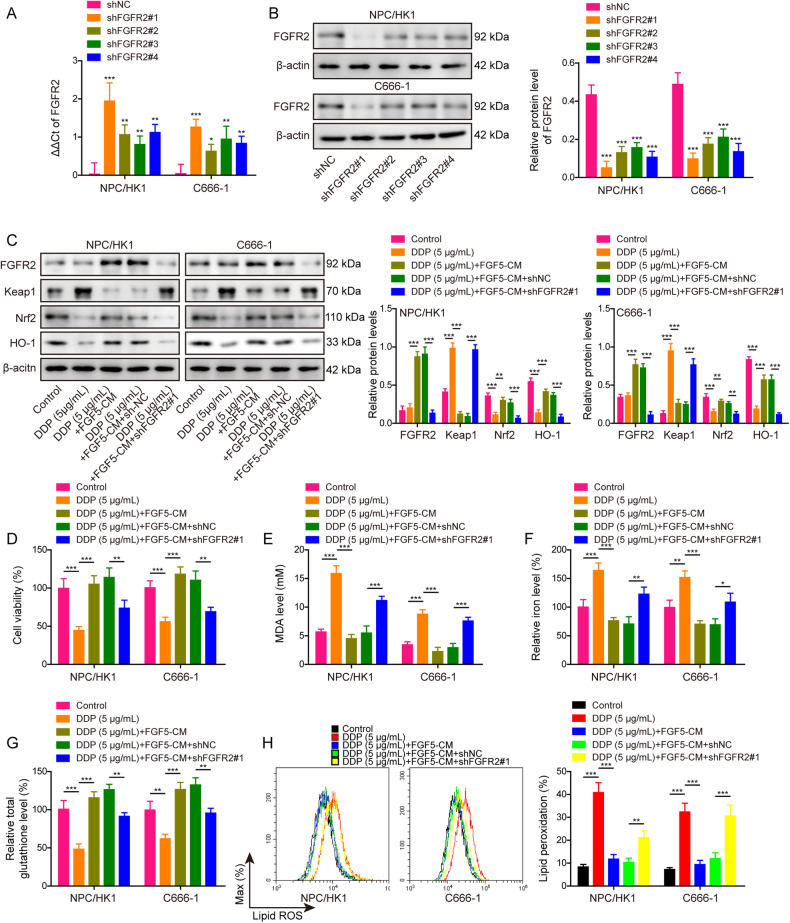
CAFs inhibit DDP-induced ferroptosis via FGF5/FGFR2 axis. **A** The mRNA level of *FGFR2* in transfected NPC cells were detected by qRT-PCR. **B** The protein level of FGFR2 in transfected NPC cells were detected by western blot. NPC/HK1 and C666-1 cells were divided into five groups: control, DDP (5 μg/mL), DDP (5 μg/mL)+FGF5-CM, DDP (5 μg/mL) + FGF5-CM + shNC and DDP (5 μg/mL) + FGF5-CM + shFGFR2#1. **C** The protein levels of FGFR2, Keap1, Nrf2 and HO-1 in NPC cells were detected by western blot. **D** Cell viability was monitored by CCK-8 assay. **E**–**G** The levels of MDA (**E**), Fe^2+^ (**F**) and GSH (**G**) in NPC cells were measured using commercial kits. **H** NPC cells were stained with BODIPY C11 and lipid peroxidation was assessed by flow cytometry. *P < 0.05, **P < 0.01, and ***P < 0.001.

As anticipated, transfection of shFGFR2 led to the reduction of FGFR2 in NPC/HK1 and C666-1 cells, and shFGFR2#1 with the highest silencing efficacy was used for the functional experiments (Fig. 7A, B). Functional studies were next carried out to investigate the role of FGF5/FGFR2 axis on ferroptosis. NPC/HK1 and C666-1 cells with FGFR2 silence were treated with DDP and CM derived from FGF5-overexpressing CAFs. Western blot results showed that CM of FGF5-overexpressing CAFs induced the protein level of FGFR2 and activated Keap1/Nrf2/HO-1 signaling, whereas silence of FGFR2 successfully reversed the above effects (Fig. 7C). In line with these findings, FGF5-overexpressing CAFs-CM protected against the decreased cell viability caused by DDP, while FGFR2 silence abrogated this protective effect in comparison with shNC (Fig. 7D). Lack of FGFR2 also attenuated the rescued effects of FGF5-overexpressing CAFs-CM on DDP-induced changes of MDA (Fig. 7E), Fe^2+^ (Fig. 7F), GSH (Fig. 7G) and lipid peroxidation (Fig. 7H) in NPC cells. These findings suggest that FGF5/FGFR2 axis contributes to the protective effects of CAFs on DDP-induced ferroptosis.

### FGF5 suppresses DDP-induced ferroptosis in NPC xenograft model

**Fig. 8:**
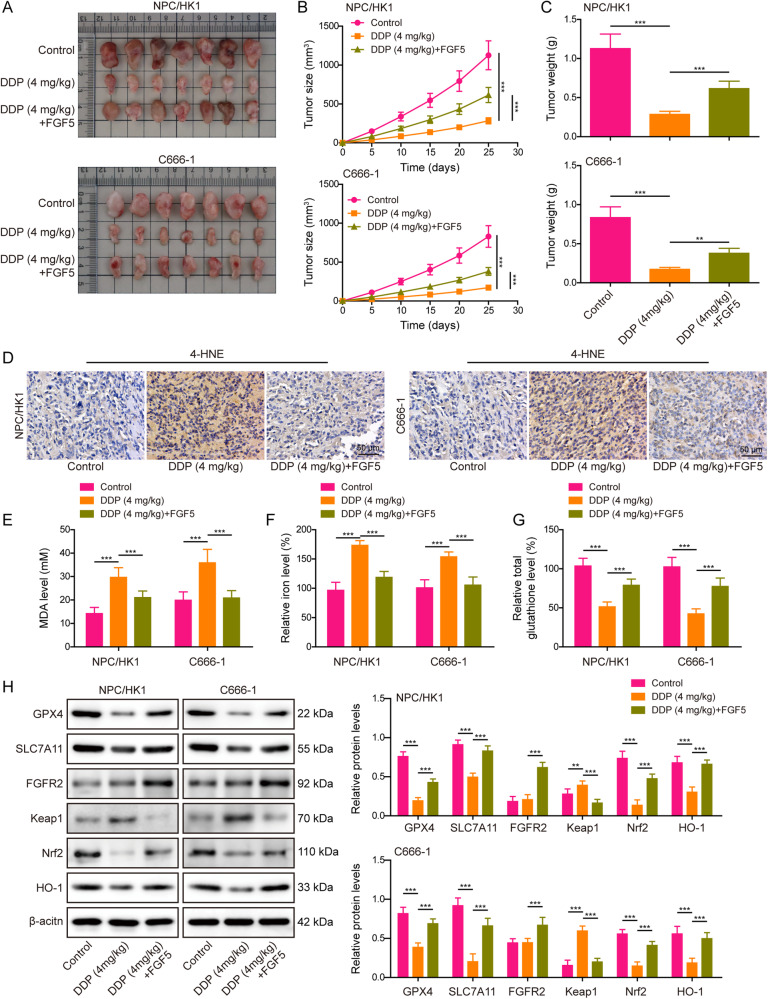
FGF5 suppresses DDP-induced ferroptosis in NPC xenograft model. Male BALB/c nude mice were randomly divided into three groups: control, DDP (4 mg/kg) and DDP (4 mg/kg)+FGF5 recombinant protein groups. **A** Representative photos of xenograft tumors. **B**, **C** Quantitative analyses of tumor volume (**B**) and weight (**C**). **D** The immunoreactivity of 4-HNE in tumors was detected by IHC analysis. Scale bar: 5 μm. **E**–**G** The levels of MDA (**E**), Fe^2+^ (**F**) and GSH (**G**) in xenograft tumors were measured using commercial kits. **H** The protein levels of GPX4, SLC7A11, FGFR2, Keap1, Nrf2 and HO-1 were detected by western blot. *P < 0.05, **P < 0.01, and ***P < 0.001.

We next validated the in vitro findings in NPC xenograft model. As presented in Fig. 8A–C, the suppressive effect of DDP on tumor growth was counteracted by FGF5 recombinant protein. Lipid peroxidation was next assessed by 4-HNE staining, and FGF5 recombinant protein attenuated the increased 4-HNE expression induced by DDP in xenograft tumors (Fig. 8D). In accordance with the in vitro data, FGF5 recombinant protein also decreased the levels of MDA (Fig. 8E) and Fe^2+^ (Fig. 8F), and increased the GSH level (Fig. 8G) induced by DDP. Western blot further revealed that DDP decreased GPX4 and SLC7A11 expression, and inactivated Keap1/Nrf2/HO-1 signaling in xenograft tumors, while these changes were abrogated in xenograft tumors derived from DDP + FGF5 group (Fig. 8H). Also, DDP treatment exerted no effects on FGFR2 expression, while FGF5 recombinant protein markedly increased FGFR2 expression in xenograft tumors (Fig. 8H). Together, these data suggest that FGF5 suppresses DDP-induced ferroptosis in vivo.

## Discussion

Chemoresistance is a major challenge for NPC treatment, and it could be attributed to various factors, such as drug efflux, death escaping, enhancement of DNA repair, Epstein-Barr virus (EBV) infection, and regulation by exosomes or non-coding RNAs [5]. Chemoresistance in NPC patients leads to poor prognosis, relapse and metastasis. Currently, the combination therapy with multidrug is one of treatments for the recurrent NPC [25], and enhanced chemosensitivity will improve the clinical outcomes of NPC patients [26]. In the present study, we demonstrated that CAFs-derived FGF5 bound FGFR2 in NPC cells, thus decreasing DDP sensitivity by suppressing ferroptosis via the activation of Keap1/Nrf2/HO-1 signaling.

DDP-based concurrent chemoradiotherapy has been used as a primary treatment in NPC [4]. Recent clinical trials have revealed that Gemcitabine and DDP induction chemotherapy improves overall survival in NPC patients [27]. Earlier studies have demonstrated that MAD2 contributes to chemosensitization to DDP in NPC cells via inducting mitotic cell death [28]. Besides previously reported DDP-induced mitotic cell death in NPC, another study showed DDP decreased cell viability that was accompanied with lipid peroxidation accumulation [19], but the detailed mechanism was not studied in this study. We found that DDP induced ferroptosis by detecting the levels of GPX4, SLC7A11, MDA, Fe^2+^, GSH and lipid peroxidation. More importantly, these changes induced by DDP were attenuated by ferroptosis inhibitor Ferrostatin-1, indicating that ferroptosis contributed to DDP-induced NPC cell death in vitro and in vivo. Our findings firstly provided more convincing evidence which supported that DDP induced ferroptosis in NPC.

Despite a number of studies have supported the tumor suppressive role of DDP in NPC, patients with DDP resistance cannot benefit from DDP therapy [5]. In recent years, many studies have reported different mechanisms underlying DDP resistance in NPC. For instance, ER-stressed NPC cells potentiated DDP resistance by modulating apoptosis and pyroptosis via releasing exosomal ERp44 [29]. LncRNA *KCNQ1OT1* suppressed the chemosensitivity to DDP via *miR-545*/*USP47* axis in NPC cells [30]. In this study, CAFs-secreted FGF5 was identified as another key player in modulating DDP resistance in NPC cells. The high level of FGF5 was observed in CAFs and CM from CAFs. In addition, DDP-induced ferroptosis was rescued by CAFs-CM, and these effects were neutralized by anti-FGF5 antibody, suggesting that endogenous FGF5 in CAFs-CM plays a critical role in this process and the effects of FGF5 in CAFs were not supraphysiological. In addition to CAFs-CM, we also reported NFs-CM and even FGF5-overexpressing NFs-derived CM had no significant effects on DDP-induced ferroptosis, because this concentration of FGF5 is quite low and not enough to suppress ferroptosis. We demonstrated for the first time that CAFs-secreted FGF5 was implicated in impairment of DDP sensitivity in NPC. We next unraveled the mechanism underlying the protective effects of CAFs-secreted FGF5 on DDP-mediated ferroptosis. Keap1-mediated proteasomal degradation regulates Nrf2 expression, and Nrf2 suppresses lipid peroxidation through inducing its downstream targets such as HO-1 [31]. The involvement of Nrf2 signaling pathway in DDP resistance has been observed in other cancers [21, 22]. In the present study, we showed CAFs-derived FGF5 protected against DDP-induced ferroptosis via activating Keap1/Nrf2/HO-1 signaling. However, whether FGF5/FGFR2 axis activates Keap1/Nrf2/HO-1 signaling directly or indirectly remains uninvestigated in this study, which needs further in-depth investigation in the future study. Moreover, the number of CAFs in radioresistant NPC tissues is more than that in radiosensitive NPC tissues, and CAFs contributes to radioresistance via IL-8/NF-κB pathway [9]. We further illustrated that CAFs not only contributed to radioresistance, but also participated in DDP sensitivity in NPC. Primary human CAFs isolated from NPC tissues were used in this study. Due to lack of cell line of CAFs, a number of studies also investigated the biological functions of CAFs only using primary CAFs in cancers [8–11, 32, 33]. Further validation of the results in CAFs cell line will strengthen the conclusion.

We next sought to identify the receptor of FGF5. FGFRs, including FGFR1-4, belong to a family of receptor tyrosine kinases [34]. Upon binding to FGF, FGFR2 undergoes conformational changes and autophosphorylation, thereby activating a variety of downstream signalings [35]. Upregulation of FGFR2 has been observed in several cancers, including breast, gastric cancer and NPC [15, 36, 37]. Recently, exon 18-truncated *FGFR2* has been reported as a therapeutic target in multiple cancers [38]. Silencing of FGFR2 facilitates DDP-induced apoptosis in NPC cells by regulating cleaved caspase-3, Bax and Bcl-2 [15]. We also demonstrated the elevation of FGFR2 in NPC tissues and cells. Among the four FGFRs, FGF5 bound to FGFR2 more tightly than other FGFRs in NPC tissues, indicating that FGFR2 activation might play a crucial role in inhibition of DDP sensitivity by CAFs. The functions of other FGFRs need further investigation in the future study. Functional experiments further confirmed the importance of FGF5/FGFR2 signaling in the regulation of DDP sensitivity by CAFs. In addition to apoptosis, we reported for the first time that FGF5/FGFR2 axis from CAFs contributed to suppressing DDP sensitivity in NPC via modulating ferroptosis.

In conclusion, we demonstrated that CAFs secrete FGF5 to inhibit ferroptosis and DDP sensitivity in NPC by regulating FGFR2/Nrf2 signaling pathway. Targeting FGF5/FGFR2/Nrf2 signaling pathway probably augments the efficacy and effective dose of DDP for NPC treatment.

## Materials and methods

### Clinical specimen

A cohort of 12 patients with NPC from 2020 to 2022 in Hunan Cancer Hospital and the Affiliated Cancer Hospital of Xiangya School of Medicine, Central South University was recruited to this study. All diagnoses were validated by pathological examination. Patients who received radio- or chemotherapy before surgery, or presented other malignancies were excluded from this study. Written consents were acquired from all participants. NPC tissues and their normal counterparts were collected during surgery. All tumor samples were stored at -80°C before use. The procedure of patient recruitment, specimen collection, and tissue processing was approved by the Ethics Committee of Hunan Cancer Hospital and the Affiliated Cancer Hospital of Xiangya School of Medicine, Central South University.

### Isolation and characterization of primary fibroblasts

As described in previous study, human CAFs and NFs were isolated from NPC tissues and their normal counterparts, respectively [9]. In brief, fresh NPC or normal tissues were cut into 2 ~ 3 mm pieces and maintained in DMEM (Gibco, Grand Island, NY, USA) containing with 10% FBS (Gibco) and 1% penicillin/streptomycin (Invitrogen, Carlsbad, CA, USA) at 37 °C and 5% CO_2_. After about 7 days, fibroblasts appeared. Primary fibroblasts were characterized by immunofluorescence and western blot using the antibodies against α-SMA, vimentin and FAP as previously described [39, 40].

### Immunofluorescence

CAFs were fixed with 4% paraformaldehyde and permeabilized with Triton X-100. After blocking with 5% BSA, CAFs were then incubated with anti-α-SMA (1:200, ab124964, Abcam, Cambridge, UK) or anti-FAP (1:100, PA5-99313, Invitrogen) antibody at 4 °C overnight. This is followed by the incubation with Alexa Fluor 488- or Alexa Fluor 594-conjugated secondary antibody (A-11008 or A-11012, Invitrogen) for 2 h at room temperature. Nuclei were visualized by DAPI staining. Images were acquired under a confocal microscope (Nikon, Tokyo, Japan).

### Cell culture, treatment and transfection

Human NPC cell lines NPC/HK1 and C666-1 cells, and normal nasopharyngeal epithelial cell line N69 cells were obtained from the Cell Bank of the Chinese Academy of Sciences (Shanghai, China). Cells were grown in RPMI 1640 (Gibco) containing 10% FBS at 37 °C and 5% CO_2_ incubator. Cells were free of mycoplasma contamination and authenticated by short tandem repeat (STR) profiling. Cisplatin (DDP, 2.5, 5, 10 μg/mL, C2210000) and Ferrostatin-1 (10 μM, SML0583) were both ordered from Sigma-Aldrich (St. Louis, MO, USA). For the ferroptosis inhibition study, NPC cells were pre-treated with 10 μM Ferrostatin-1 for 2 h, followed by the treatment of 5 μg/mL DDP for 48 h. For the neutralization study, cells were treated with FGF5 neutralizing antibody (10 ng/mL, AF-237-NA, R&D Systems, Minneapolis, MN, USA) for 48 h.

The lentivirus vectors used to silence or overexpress FGF5, FGFR2 and Nrf2 were constructed from GenePharma (Shanghai, China). The lentivirus were transfected into the HEK-293T cells with helper vectors pMD2G and pSPAX2 using Lipofectamine 2000 reagent (Invitrogen), then lentiviral particles were mixed with polybrene (5 µg/mL, TR1003, Sigma-Aldrich) and added into the CAFs or NPC cells with multiplicity of infection (MOI) of 10. The sequences of shRNAs were shown in Supplemental materials.

### Preparation of conditioned medium (CM)

NFs or CAFs were cultured in DMEM containing 10% FBS at 37 °C overnight. Cells were then refreshed with serum-free medium and then the cell culture supernatants were collected after culturing 48 h. Cell pellets were removed by centrifugation, and CM was harvested to incubate NPC cells for subsequent experiments.

### CCK-8 assay

Cell viability of NPC cells was monitored using a CCK-8 Kit (Beyotime, Shanghai, China). Briefly, treated NPC/HK1 and C666-1 cells were incubated with CCK-8 solution (10 μL per well) for 1 h, and A450 was assessed using a microplate reader (BioTek, Winooski, VT, USA).

### Measurement of MDA, GSH and ferrous iron

The levels of MDA, GSH and ferrous iron in the homogenate tissues and cell lysates were evaluated by the Lipid Peroxidation Assay Kit (ab118970, Abcam), Glutathione Assay Kit (CS0260, Sigma-Aldrich), and Iron Assay Kit (MAK025, Sigma-Aldrich), respectively. The specific procedures were performed according to guidelines of the above kits.

### Measurement of lipid peroxidation

Lipid peroxidation was assessed using BODIPY 581/591 C11 Kit (D3861, Invitrogen) as described previously [41]. Cells were stained with 5 μM BODIPY C11 at 37 °C for 1 h, and analyzed by flow cytometry (BD Biosciences).

### ELISA assay

The protein level of FGF5 in culture medium secreted from CAFs or NFs was detected using Human FGF5 ELISA Kit (EH191RB, Invitrogen) according to the protocol.

### Co-immunoprecipitation (Co-IP)

Tumor tissues were lysed with cell lysis buffer for western blot and IP (P0013, Beyotime), and incubated with anti-FGF5 antibody (0.5 μL per mg lysates, PA5-80630, Invitrogen) or normal rabbit IgG (ab172730, Abcam) at 4 °C overnight. Protein complexes were then enriched by Protein A/G agarose (Pierce, Rockford, IL, USA) at 4 °C for 4 h, and the protein complexes were eluted. The immunoprecipitated FGFR1, FGFR2, FGFR3, FGFR4 and FGF5 were analyzed by western blot.

### Xenograft study

Male BALB/c nude mice (6 week-old, *n* = 7 per group) were ordered from SLAC Laboratory Animal Center (Shanghai, China). All animal studies were approved by Hunan Cancer Hospital and the Affiliated Cancer Hospital of Xiangya School of Medicine, Central South University. The investigators were blinded to the group allocation during the experiment. To study the anti-tumor effects of DDP in vivo, mice were randomly divided into five groups: control, DDP (1 mg/kg), DDP (2 mg/kg), DDP (4 mg/kg) and DDP (4 mg/kg)+Ferrostatin-1 (10 mg/kg). To investigate the effect of FGF5, mice were randomly divided into three groups: control, DDP and DDP + FGF5 recombinant protein groups. NPC/HK1 or C666-1 cells were subcutaneously inoculated into the flank of mice. After injection, mice were administered with different doses of DDP (1, 2, 4 mg/kg) intraperitoneally every 3 days. For DDP + Ferrostatin-1 group, mice were intraperitoneally administered with DDP (4 mg/kg) every 3 days and Ferrostatin-1 (10 mg/kg) once a week. In some experiments, mouse FGF5 recombinant protein (CSB-YP008632MO, Cusabio, Wuhan, China) were injected via tail vein on day 2. Tumor size was measured every 5 days, and tumor volume was calculated as follows: 0.5 × length (L) × width (W)^2^. Tumors were collected and weighted on day 25, followed by the IHC staining, western blot and analyses of MDA, GSH and ferrous iron.

### IHC assay

Paraffin-embedded human NPC, adjacent normal tissues and xenograft tumors were deparaffinized, rehydrated and subjected to antigen retrieval. After blocking with 10% normal goat serum, sections were incubated with anti-4-HNE (1:25, ab48506, Abcam), anti-Nrf2 (1:100, PA5-27882, Invitrogen), anti-FGF5 (1:50, PA5-67553, Invitrogen), or anti-FGFR2 (1:100, ab10648, Abcam) antibody at 4 °C overnight. This is followed by the incubation with HRP-conjugated secondary antibodies (31430 or 31460, Invitrogen). Signals were visualized using DAB Horseradish Peroxidase Color Development Kit (P0202, Beyotime). Images were acquired under a microscope. TMA of NPC and nasal polyp tissues was purchased from Tanda Biotechnology company (Wuhan, China) and stained following the IHC protocol.

### qRT-PCR

Total RNA was extracted from tissues and cells using Trizol (Invitrogen) and reversely transcribed using SuperScript IV reverse transcriptase (Invitrogen). qRT-PCR was conducted using SYBR Green MasterMix (Applied Biosystems, Foster City, CA, USA) on ABI7500 Real-Time PCR System. The expression levels of target genes were calculated using ΔΔCT method. β-actin was used as an internal control.

### Western blot

Protein lysates were prepared using RIPA lysis buffer (Beyotime), and quantified using BCA Protein Assay Kit (Beyotime). Proteins were separated by SDS-PAGE and transferred onto nitrocellulose membranes. Blots were then blocked with 5% non-fat milk and incubated with primary antibodies at 4 °C overnight, followed by the incubation with secondary antibody (31430 or 31460, Invitrogen). Signals were detected using ECL substrate (Beyotime). Primary antibodies used in western blot as follows: anti-GPX4 (1:1000, ab125066, Abcam), anti-SLC7A11 (1:1000, ab175186, Abcam), anti-α-SMA (1:2000, ab124964, Abcam), anti-vimentin (1:1000, ab92547, Abcam), anti-FAP (1:2000, PA5-99313, Invitrogen), anti-FGF5 (1:1000, PA5-80630, Invitrogen), anti-Keap1 (1:3000, ab119403, Abcam), anti-Nrf2 (1:2000, PA5-27882, Invitrogen), anti-HO-1 (1:3000, ab68477, Abcam), anti-FGFR1 (1:500, ab76464, Abcam), anti-FGFR2 (1:1000, ab10648, Abcam), anti-FGFR3 (1:1000, ab133644, Abcam), anti-FGFR4 (1:1000, ab178396, Abcam) and anti-β-actin (1:2000, ab8226, Abcam) antibodies. Uncropped western blots were shown in Supplemental materials.

### Statistical analysis

All cell experiments were performed in at least three biological replicates, and each biological replicate contained three technical replicates. Data were analyzed using GraphPad Prism 7.0 software and presented as mean ± standard deviation (SD). All the data meet the assumption of normal distribution. Differences between two groups was examined using Student’s *t*-test and those from more than two groups using one-way analysis of variance (ANOVA) followed by Tukey post hoc test. *P* < 0.05 was considered statistically significant.

## Availability of data and material

All data generated or analysed during this study are included in this published article. Supplemental figures, uncropped western blots, and sequences of shRNAs were shown in Supplemental materials.

### Supplementary information

Supplemental materials

Original Data File
