# Supplementary material for: Cancer-associated fibroblasts secrete FGF5 to inhibit ferroptosis to decrease cisplatin sensitivity in nasopharyngeal carcinoma through binding to FGFR2
Source: Cell Death Dis. 2024 Apr 18;15(4):279. doi: 10.1038/s41419-024-06671-0 (PMC11026472; doi:10.1038/s41419-024-06671-0)
Supplement: Supplementary file 1 — Supplemental materials [file 41419_2024_6671_MOESM1_ESM.docx]

**Supplemental figures：**

**
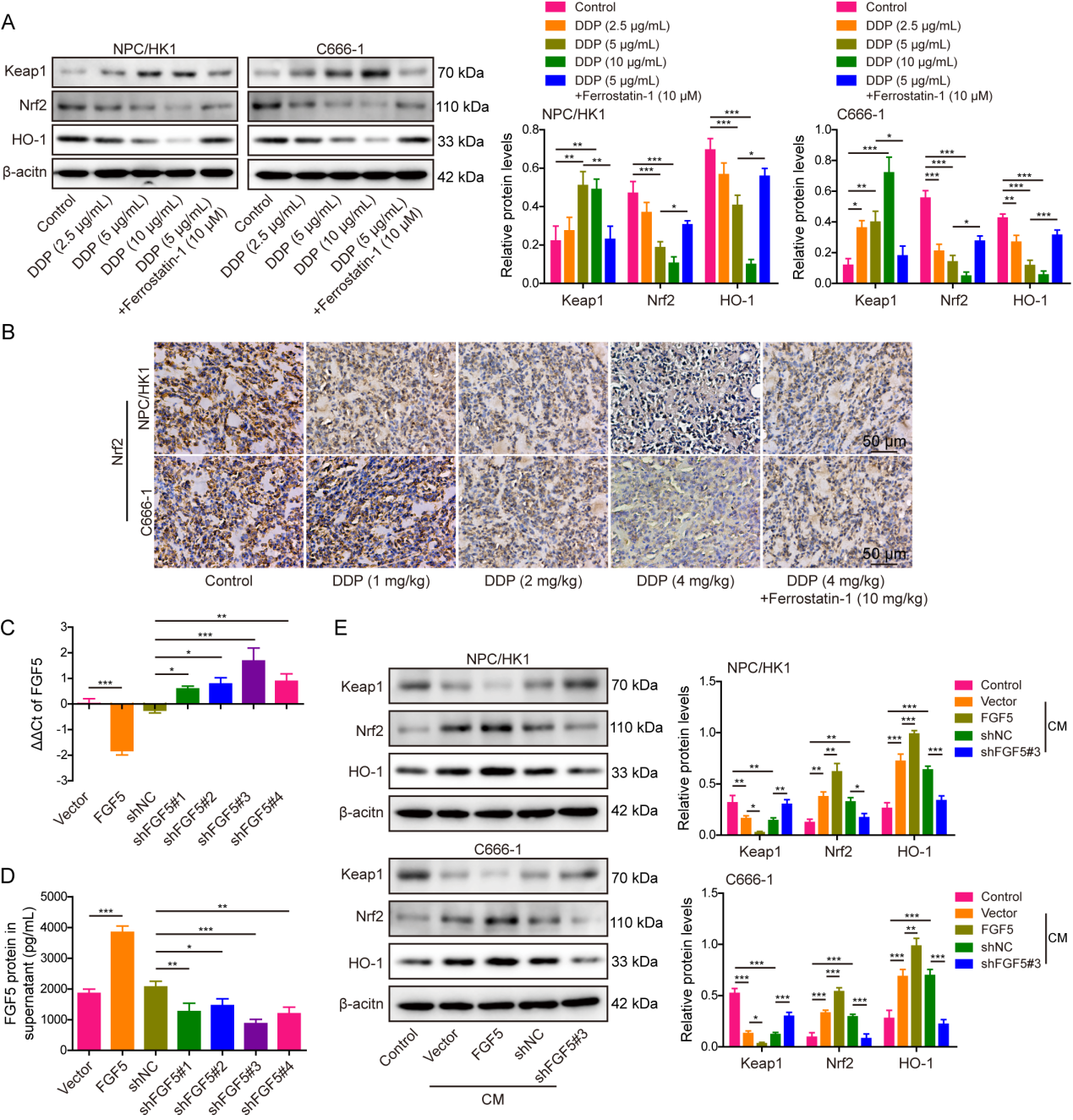
**

**Figure S1. CAFs-secreted FGF5 activates Keap1/Nrf2/HO-1 pathway in NPC cells.**

(A) The protein levels of Keap1, Nrf2 and HO-1 in NPC cells were detected by western blot. (B) The immunoreactivity of Nrf2 in xenograft tumors was detected by IHC analysis. Scale bar: 50 μm. (C) The mRNA level of *FGF5* in CAFs was detected by qRT-PCR. (D) The secretion of FGF5 in culture medium of CAFs was assessed by ELISA assay. (E) The protein levels of Keap1, Nrf2 and HO-1 in NPC cells were detected by western blot. **P*<0.05, ***P*<0.01, and ****P*<0.001.

**
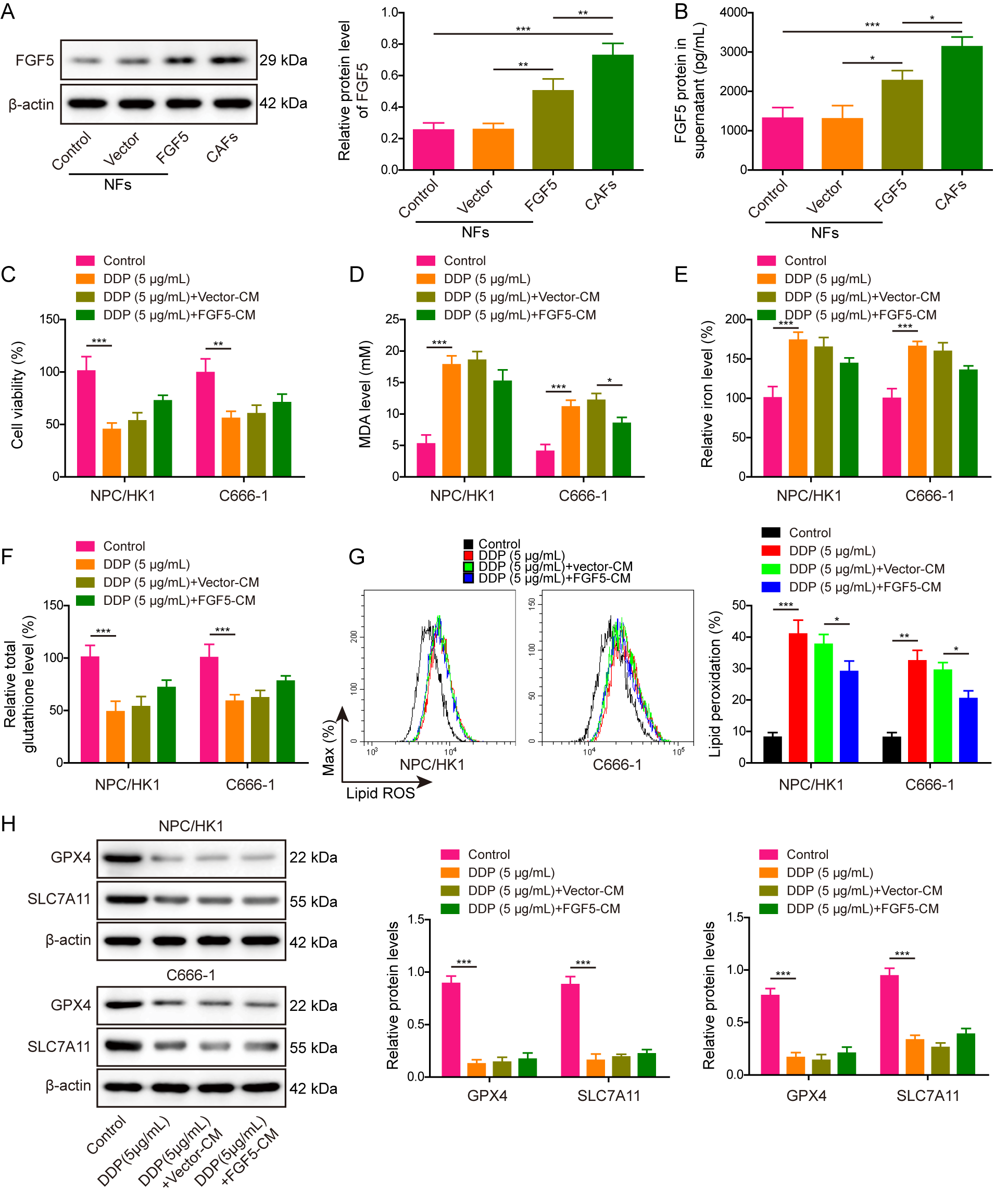
**

**Figure S2. Effects of NFs on ferroptosis of NPC cells caused by DDP.**

(A) The protein level of FGF5 in NFs or CAFs was detected by western blot. (B) The secretion of FGF5 by NFs or CAFs was assessed by ELISA assay. NPC/HK1 and C666-1 cells were divided into four groups: control, DDP (5 μg/mL), DDP (5 μg/mL)+Vector-CM, DDP (5 μg/mL)+FGF5-CM. (C) Cell viability was monitored by CCK-8 assay. (D-F) The levels of MDA (D), Fe^2+^ (E) and GSH (F) in NPC cells were measured using commercial kits. (G) NPC cells were stained with BODIPY C11 and lipid peroxidation was assessed by flow cytometry. (H) The protein levels of GPX4 and SLC7A11 were detected by western blot. *P<0.05, **P<0.01, and ***P<0.001.

**Uncropped western blots:**

**
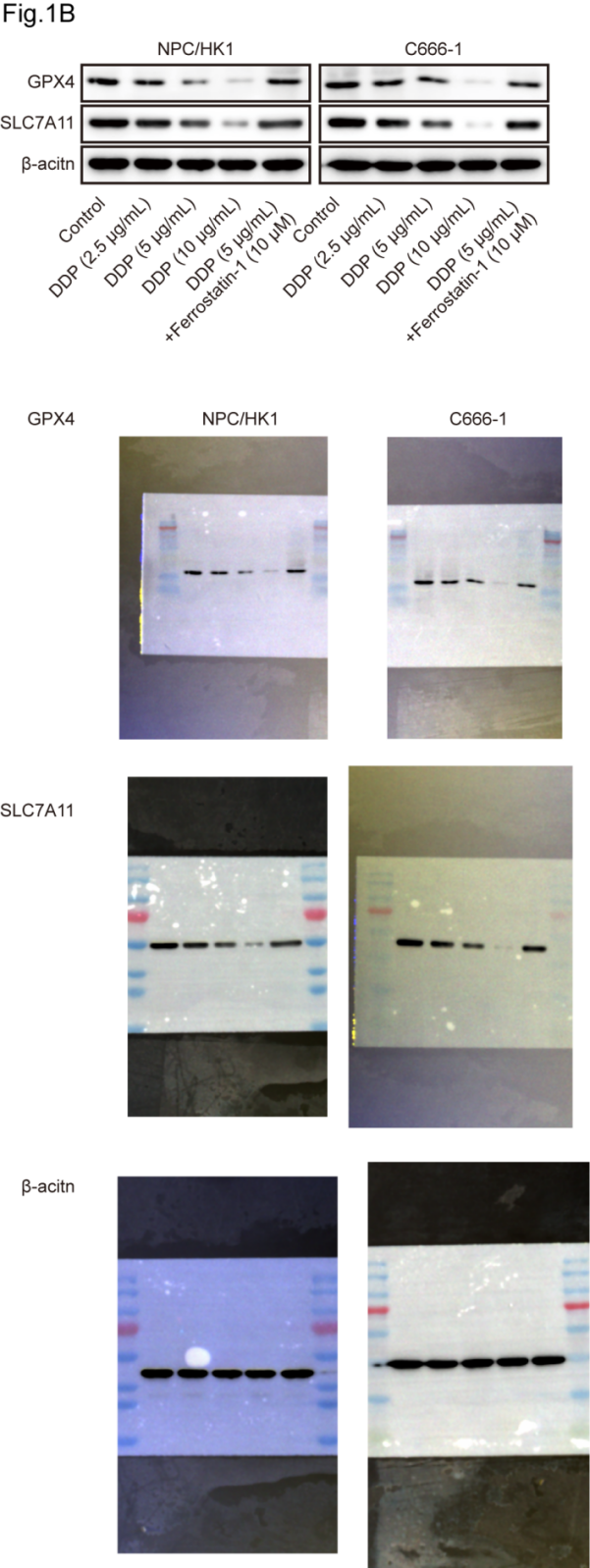
**

**
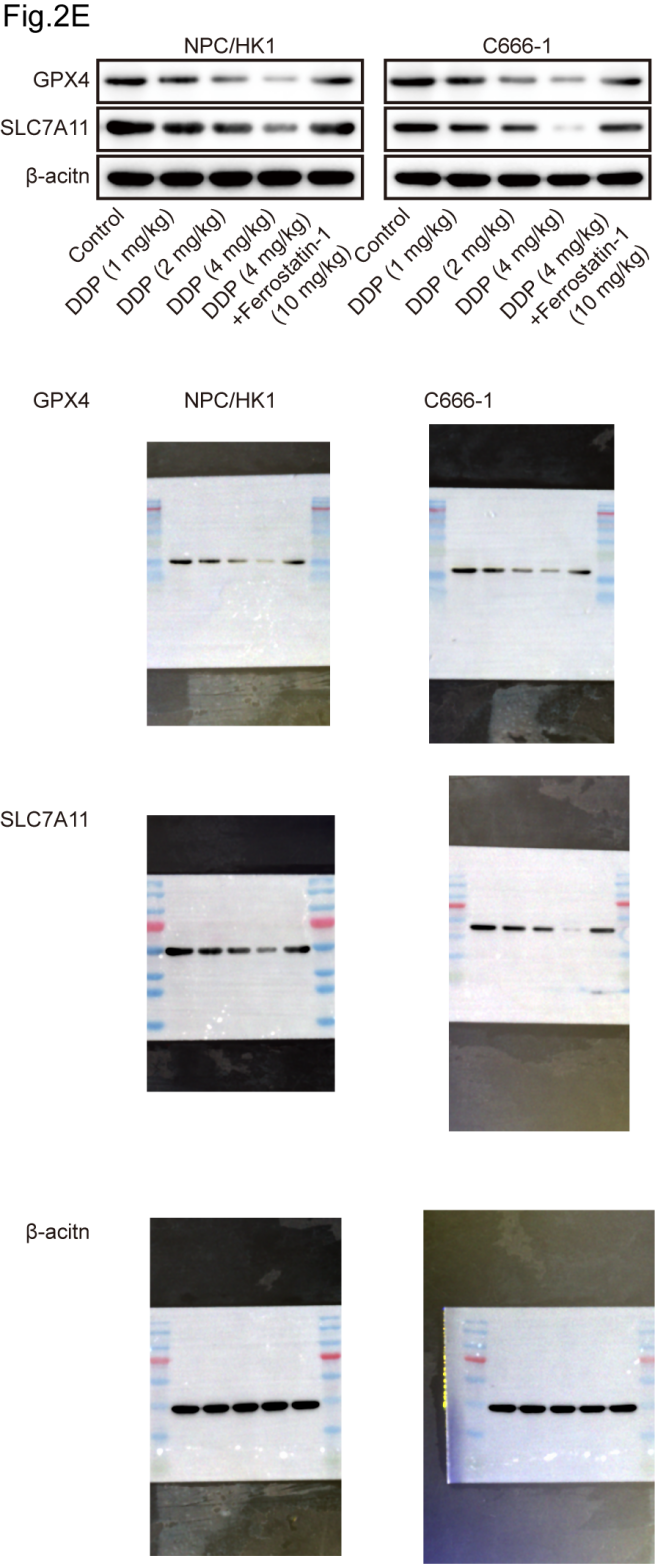
**

**
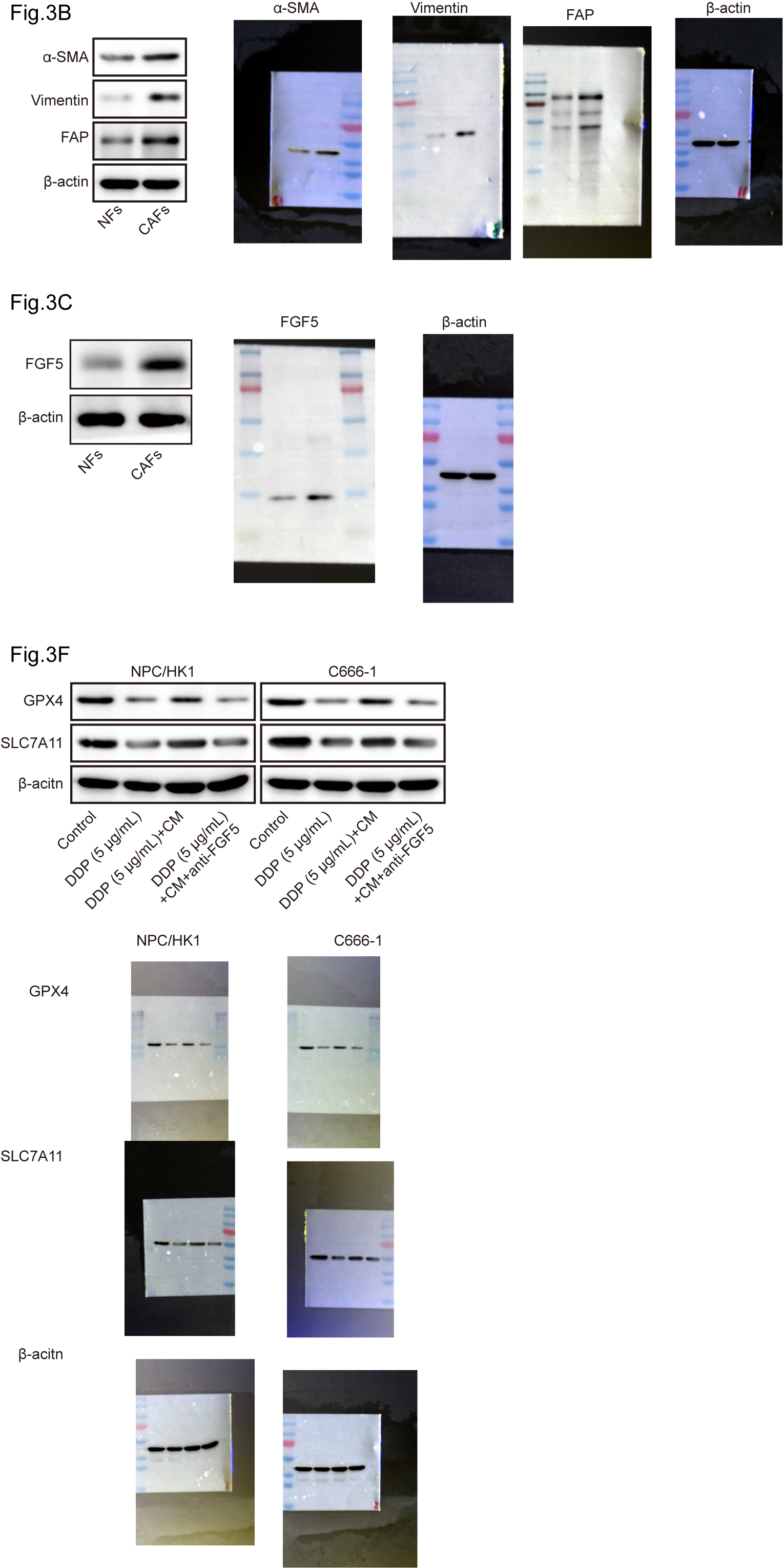
**

**
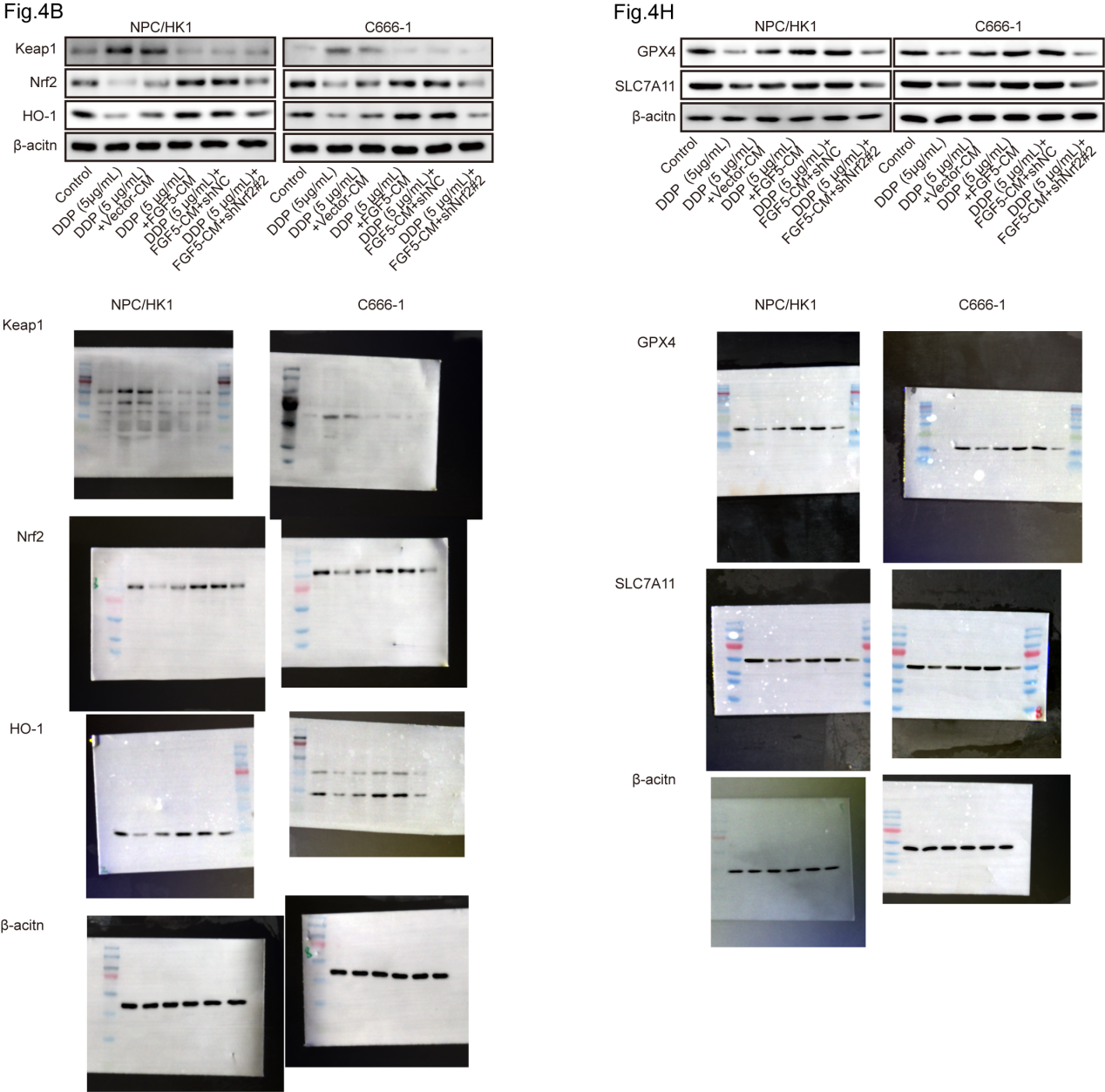
**

**
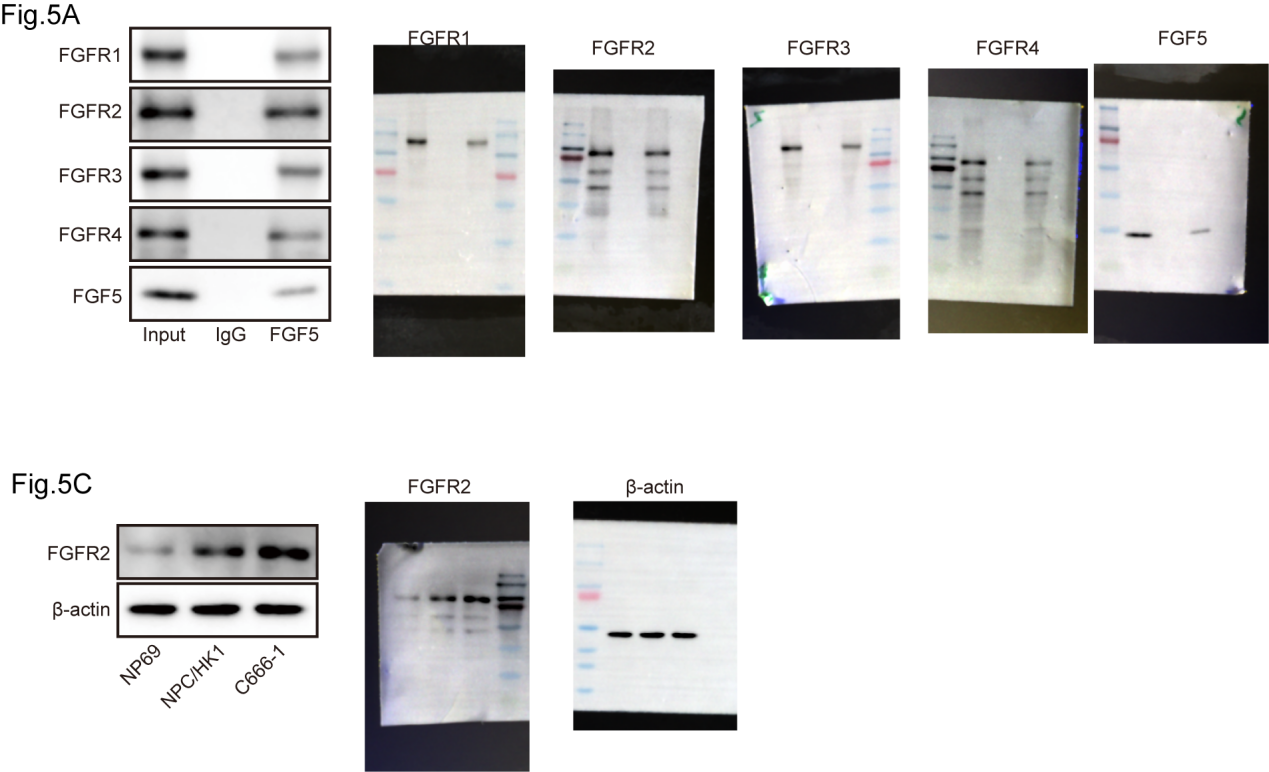
**

**
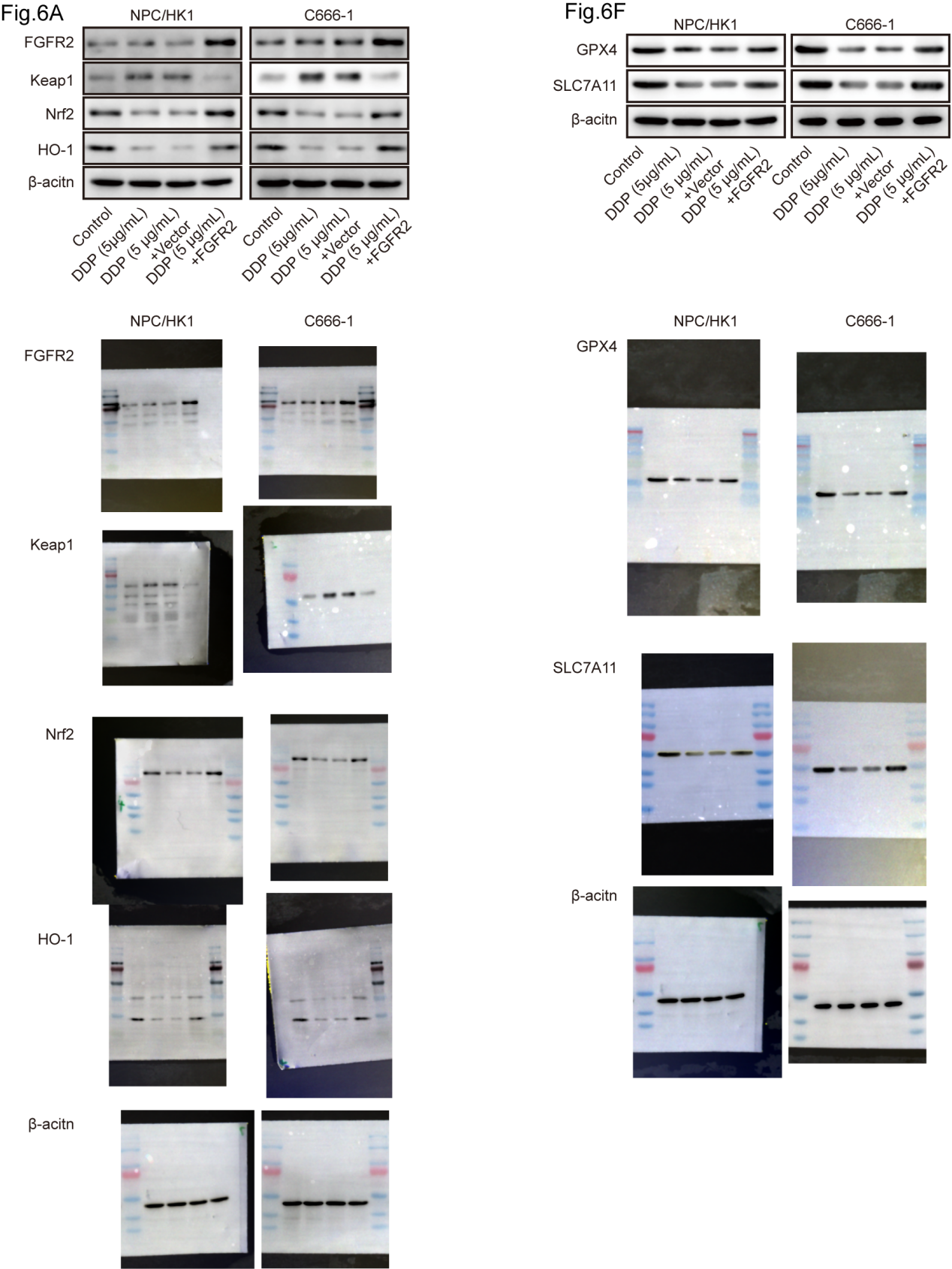
**

**
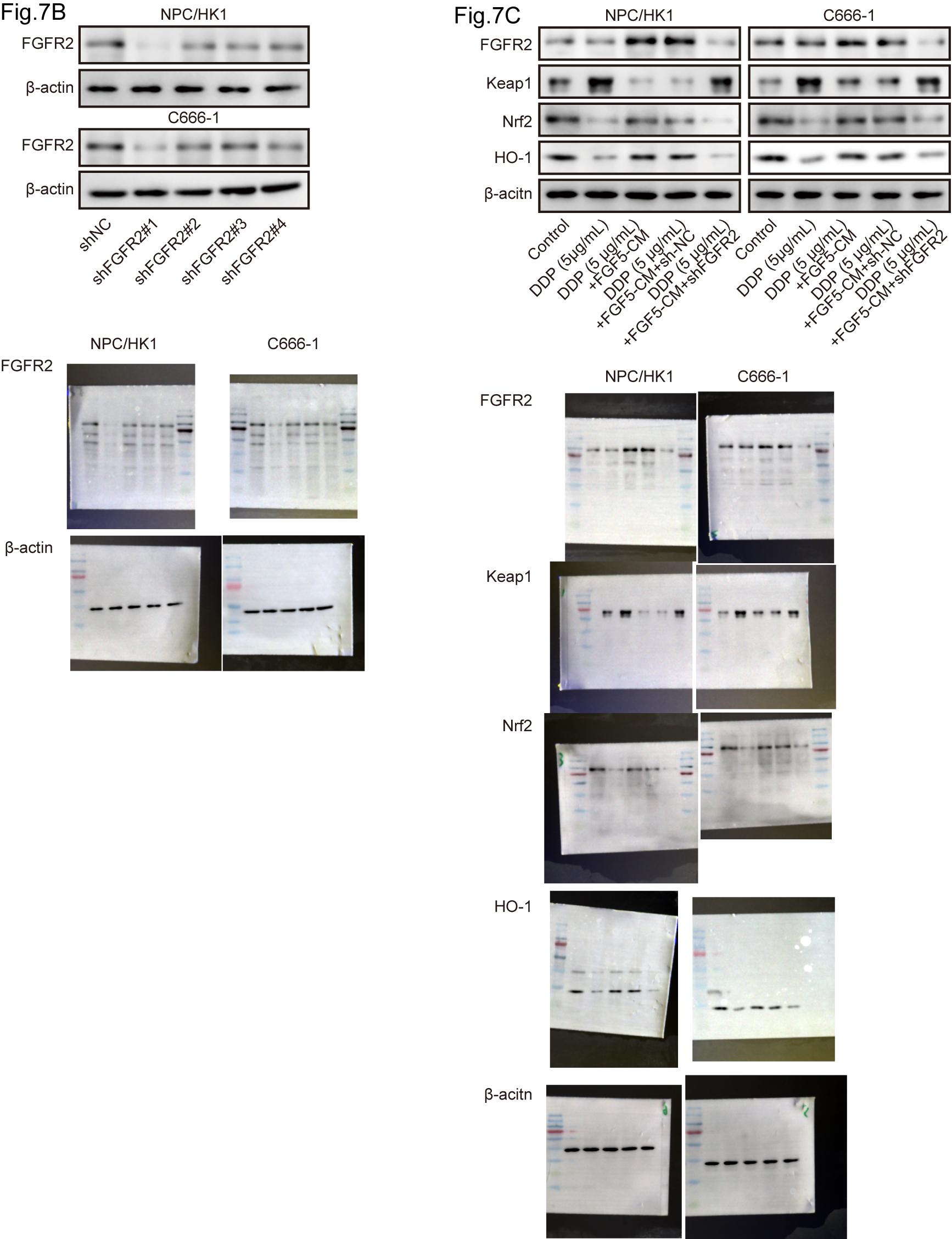
**

**
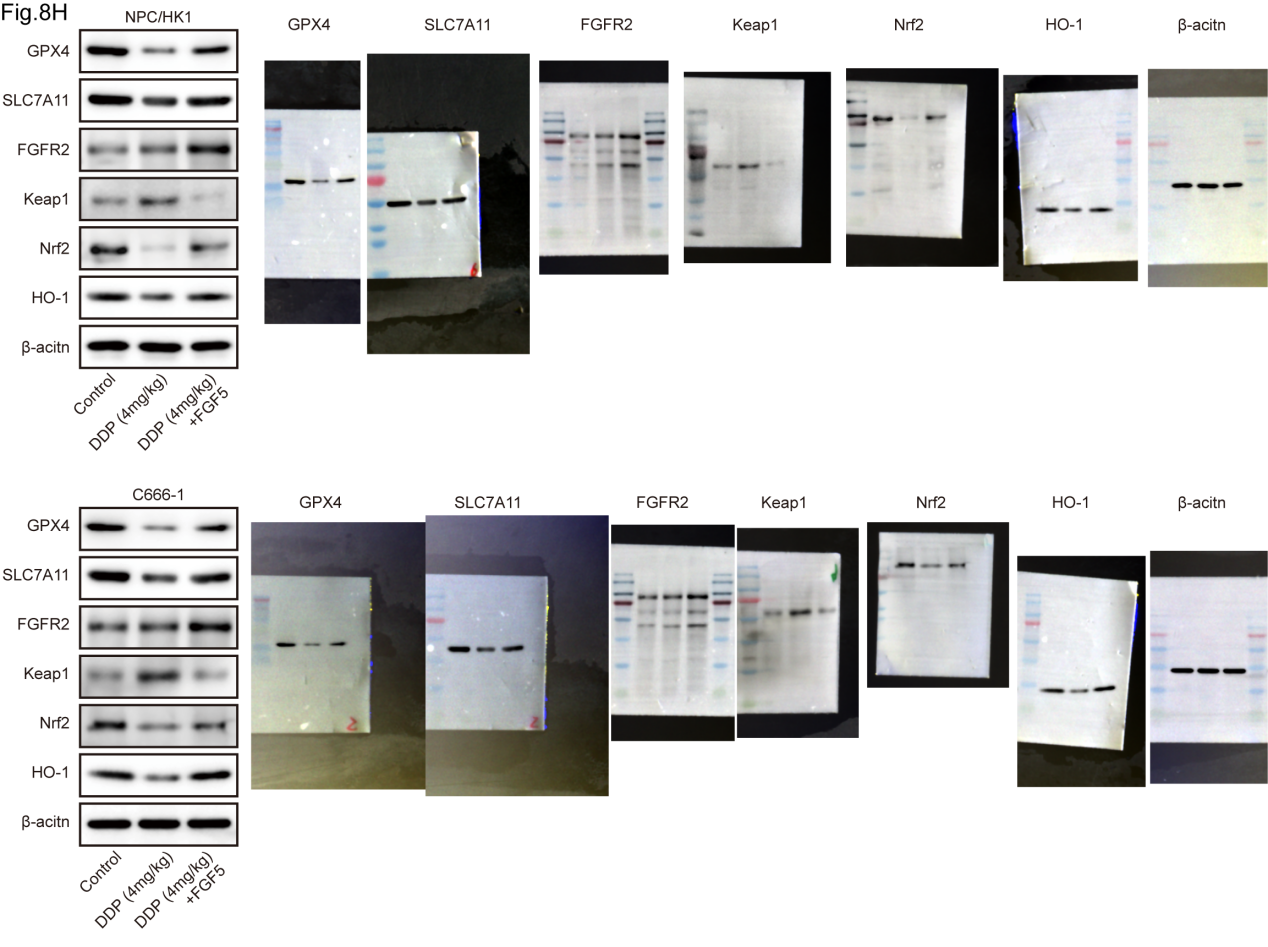
**

**
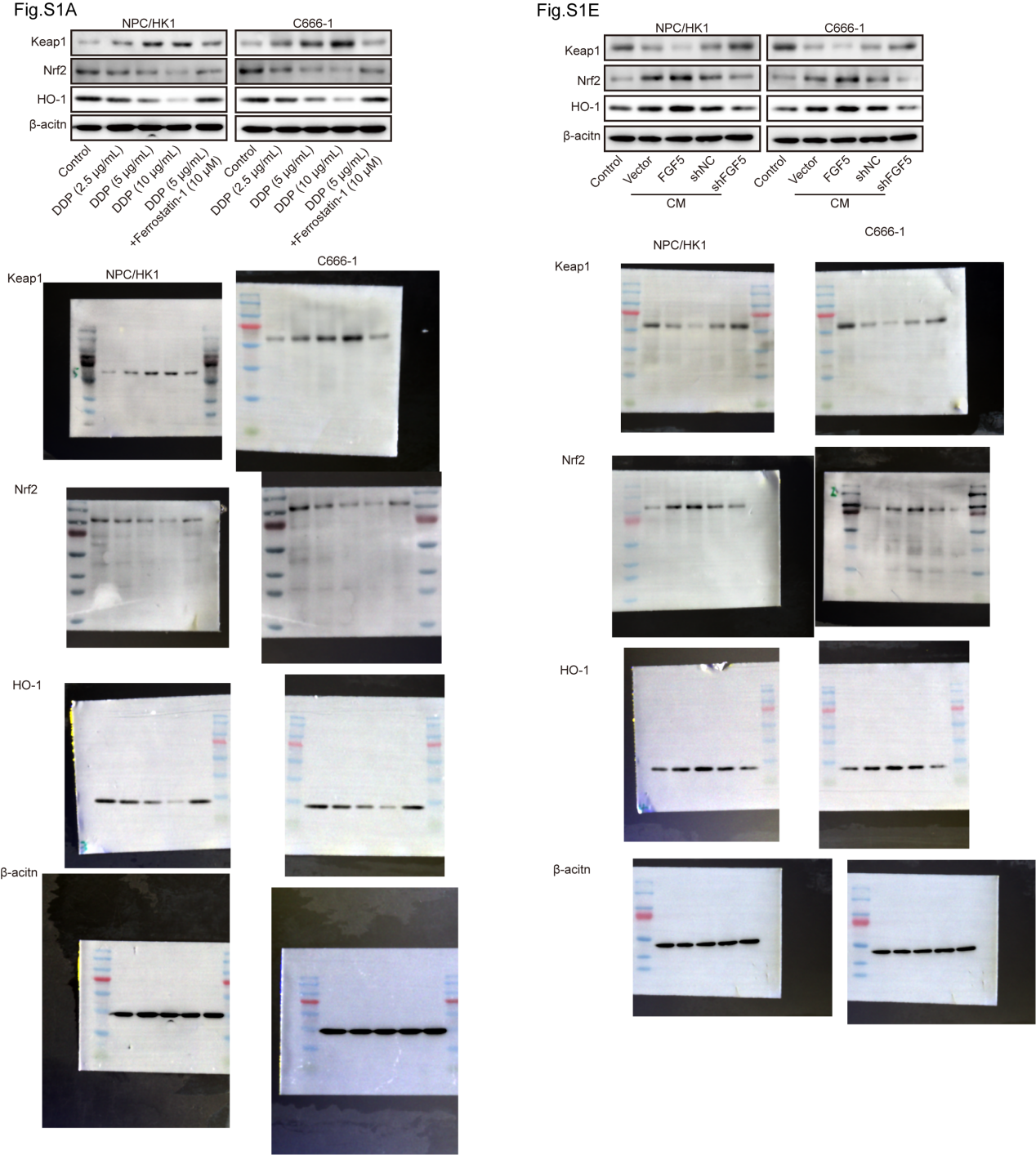
**

**
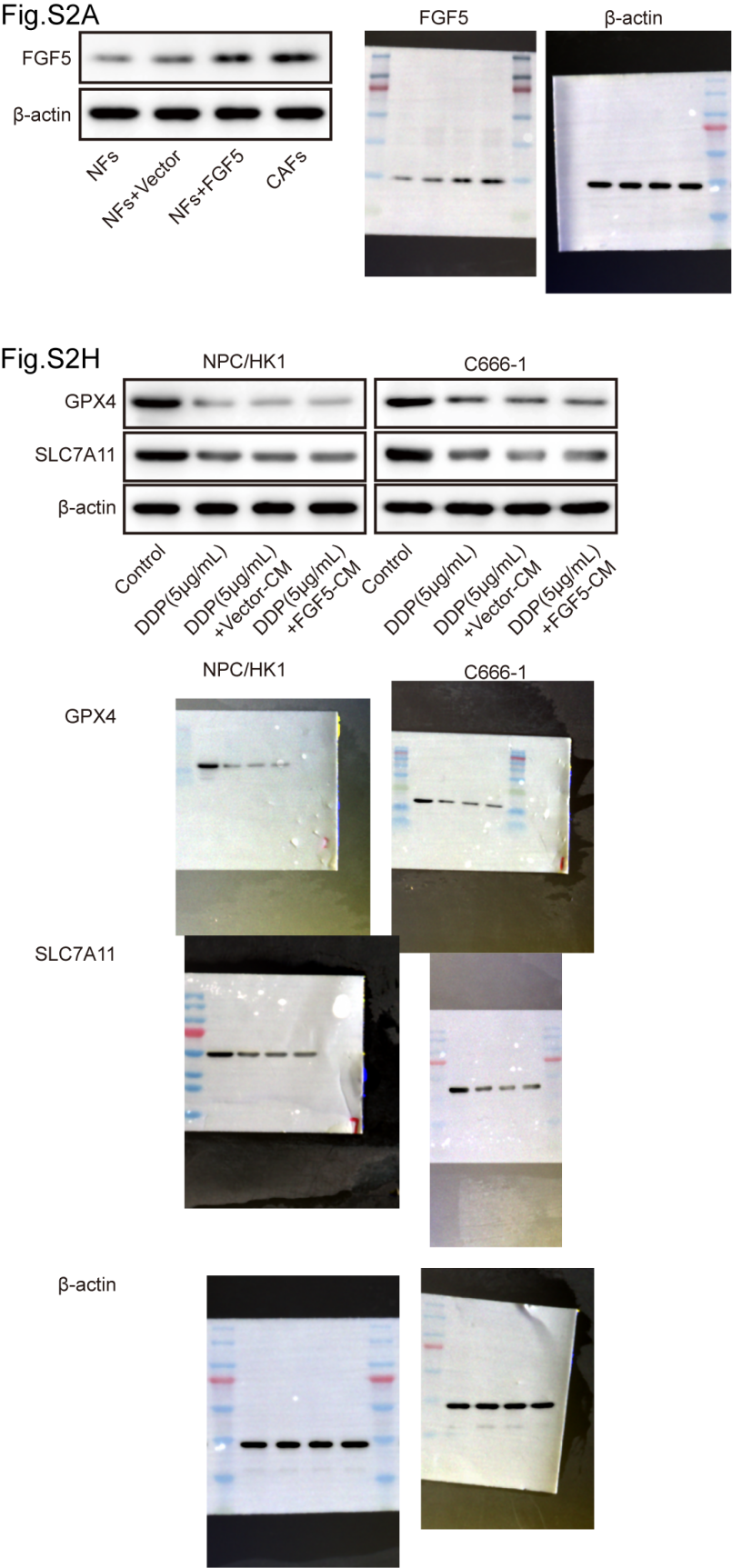
**

**Sequences of shRNAs：**

# **Homo sapiens fibroblast growth factor 5 (FGF5), transcript variant 1, mRNA**

NCBI Reference Sequence: NM_004464.4

atgagcttgtccttcctcctcctcctcttcttcagccacctgatcctcagcgcctgggctcacggggagaagcgtctcgcccccaaagggcaacccggacccgctgccactgataggaaccctagaggctccagcagcagacagagcagcagtagcgctatgtcttcctcttctgcctcctcctcccccgcagcttctctgggcagccaaggaagtggcttggagcagagcagtttccagtggagcccctcggggcgccggaccggcagcctctactgcagagtgggcatcggtttccatctgcagatctacccggatggcaaagtcaatggatcccacgaagccaatatgttaagtgttttggaaatatttgctgtgtctcaggggattgtaggaatacgaggagttttcagcaacaaatttttagcgatgtcaaaaaaaggaaaactccatgcaagtgccaagttcacagatgactgcaagttcagggagcgttttcaagaaaatagctataatacctatgcctcagcaatacatagaactgaaaaaacagggcgggagtggtatgtggccctgaataaaagaggaaaagccaaacgagggtgcagcccccgggttaaaccccagcatatctctacccattttctgccaagattcaagcagtcggagcagccagaactttctttcacggttactgttcctgaaaagaaaaagccacctagccctatcaagccaaagattcccctttctgcacctcggaaaaataccaactcagtgaaatacagactcaagtttcgctttggataa

SH1：cccagcatatctctacccatt

| S | AATTGcccagcatatctctacccattTCAAGAGaatgggtagagatatgctgggTTTTTT |
| --- | --- |
| A | GATCAAAAAAcccagcatatctctacccattCTCTTGAaatgggtagagatatgctgggC |

SH2：cctatgcctcagcaatacata

| S | AATTGcctatgcctcagcaatacataTCAAGAGtatgtattgctgaggcataggTTTTTT |
| --- | --- |
| A | GATCAAAAAAcctatgcctcagcaatacataCTCTTGAtatgtattgctgaggcataggC |

SH3：gaagccaatatgttaagtgtt

| S | AATTGgaagccaatatgttaagtgttTCAAGAGaacacttaacatattggcttcTTTTTT |
| --- | --- |
| A | GATCAAAAAAgaagccaatatgttaagtgttCTCTTGAaacacttaacatattggcttcC |

SH4：ccagaactttctttcacggtt

| S | AATTGccagaactttctttcacggttTCAAGAGaaccgtgaaagaaagttctggTTTTTT |
| --- | --- |
| A | GATCAAAAAAccagaactttctttcacggttCTCTTGAaaccgtgaaagaaagttctggC |

# **Homo sapiens fibroblast growth factor receptor 2 (FGFR2), transcript variant 1, mRNA**

NCBI Reference Sequence: NM_000141.5

atggtcagctggggtcgtttcatctgcctggtcgtggtcaccatggcaaccttgtccctggcccggccctccttcagtttagttgaggataccacattagagccagaagagccaccaaccaaataccaaatctctcaaccagaagtgtacgtggctgcgccaggggagtcgctagaggtgcgctgcctgttgaaagatgccgccgtgatcagttggactaaggatggggtgcacttggggcccaacaataggacagtgcttattggggagtacttgcagataaagggcgccacgcctagagactccggcctctatgcttgtactgccagtaggactgtagacagtgaaacttggtacttcatggtgaatgtcacagatgccatctcatccggagatgatgaggatgacaccgatggtgcggaagattttgtcagtgagaacagtaacaacaagagagcaccatactggaccaacacagaaaagatggaaaagcggctccatgctgtgcctgcggccaacactgtcaagtttcgctgcccagccggggggaacccaatgccaaccatgcggtggctgaaaaacgggaaggagtttaagcaggagcatcgcattggaggctacaaggtacgaaaccagcactggagcctcattatggaaagtgtggtcccatctgacaagggaaattatacctgtgtagtggagaatgaatacgggtccatcaatcacacgtaccacctggatgttgtggagcgatcgcctcaccggcccatcctccaagccggactgccggcaaatgcctccacagtggtcggaggagacgtagagtttgtctgcaaggtttacagtgatgcccagccccacatccagtggatcaagcacgtggaaaagaacggcagtaaatacgggcccgacgggctgccctacctcaaggttctcaaggccgccggtgttaacaccacggacaaagagattgaggttctctatattcggaatgtaacttttgaggacgctggggaatatacgtgcttggcgggtaattctattgggatatcctttcactctgcatggttgacagttctgccagcgcctggaagagaaaaggagattacagcttccccagactacctggagatagccatttactgcataggggtcttcttaatcgcctgtatggtggtaacagtcatcctgtgccgaatgaagaacacgaccaagaagccagacttcagcagccagccggctgtgcacaagctgaccaaacgtatccccctgcggagacaggtaacagtttcggctgagtccagctcctccatgaactccaacaccccgctggtgaggataacaacacgcctctcttcaacggcagacacccccatgctggcaggggtctccgagtatgaacttccagaggacccaaaatgggagtttccaagagataagctgacactgggcaagcccctgggagaaggttgctttgggcaagtggtcatggcggaagcagtgggaattgacaaagacaagcccaaggaggcggtcaccgtggccgtgaagatgttgaaagatgatgccacagagaaagacctttctgatctggtgtcagagatggagatgatgaagatgattgggaaacacaagaatatcataaatcttcttggagcctgcacacaggatgggcctctctatgtcatagttgagtatgcctctaaaggcaacctccgagaatacctccgagcccggaggccacccgggatggagtactcctatgacattaaccgtgttcctgaggagcagatgaccttcaaggacttggtgtcatgcacctaccagctggccagaggcatggagtacttggcttcccaaaaatgtattcatcgagatttagcagccagaaatgttttggtaacagaaaacaatgtgatgaaaatagcagactttggactcgccagagatatcaacaatatagactattacaaaaagaccaccaatgggcggcttccagtcaagtggatggctccagaagccctgtttgatagagtatacactcatcagagtgatgtctggtccttcggggtgttaatgtgggagatcttcactttagggggctcgccctacccagggattcccgtggaggaactttttaagctgctgaaggaaggacacagaatggataagccagccaactgcaccaacgaactgtacatgatgatgagggactgttggcatgcagtgccctcccagagaccaacgttcaagcagttggtagaagacttggatcgaattctcactctcacaaccaatgaggaatacttggacctcagccaacctctcgaacagtattcacctagttaccctgacacaagaagttcttgttcttcaggagatgattctgttttttctccagaccccatgccttacgaaccatgccttcctcagtatccacacataaacggcagtgttaaaacatga

SH1：agccctgtttgatagagtata

| S | AATTGagccctgtttgatagagtataTCAAGAGtatactctatcaaacagggctTTTTTT |
| --- | --- |
| A | GATCAAAAAAagccctgtttgatagagtataCTCTTGAtatactctatcaaacagggctC |

SH2：ttagttgaggataccacatta

| S | AATTGttagttgaggataccacattaTCAAGAGtaatgtggtatcctcaactaaTTTTTT |
| --- | --- |
| A | GATCAAAAAAttagttgaggataccacattaCTCTTGAtaatgtggtatcctcaactaaC |

SH3：gccaccaaccaaataccaaat

| S | AATTGgccaccaaccaaataccaaatTCAAGAGatttggtatttggttggtggcTTTTTT |
| --- | --- |
| A | GATCAAAAAAgccaccaaccaaataccaaatCTCTTGAatttggtatttggttggtggcC |

SH4：cagtgaaacttggtacttcat

| S | AATTGcagtgaaacttggtacttcatTCAAGAGatgaagtaccaagtttcactgTTTTTT |
| --- | --- |
| A | GATCAAAAAAcagtgaaacttggtacttcatCTCTTGAatgaagtaccaagtttcactgC |

# **Homo sapiens NFE2 like bZIP transcription factor 2 (NFE2L2), transcript variant 1, mRNA**

NCBI Reference Sequence: NM_006164.5

atgatggacttggagctgccgccgccgggactcccgtcccagcaggacatggatttgattgacatactttggaggcaagatatagatcttggagtaagtcgagaagtatttgacttcagtcagcgacggaaagagtatgagctggaaaaacagaaaaaacttgaaaaggaaagacaagaacaactccaaaaggagcaagagaaagcctttttcgctcagttacaactagatgaagagacaggtgaatttctcccaattcagccagcccagcacatccagtcagaaaccagtggatctgccaactactcccaggttgcccacattcccaaatcagatgctttgtactttgatgactgcatgcagcttttggcgcagacattcccgtttgtagatgacaatgaggtttcttcggctacgtttcagtcacttgttcctgatattcccggtcacatcgagagcccagtcttcattgctactaatcaggctcagtcacctgaaacttctgttgctcaggtagcccctgttgatttagacggtatgcaacaggacattgagcaagtttgggaggagctattatccattcctgagttacagtgtcttaatattgaaaatgacaagctggttgagactaccatggttccaagtccagaagccaaactgacagaagttgacaattatcatttttactcatctataccctcaatggaaaaagaagtaggtaactgtagtccacattttcttaatgcttttgaggattccttcagcagcatcctctccacagaagaccccaaccagttgacagtgaactcattaaattcagatgccacagtcaacacagattttggtgatgaattttattctgctttcatagctgagcccagtatcagcaacagcatgccctcacctgctactttaagccattcactctctgaacttctaaatgggcccattgatgtttctgatctatcactttgcaaagctttcaaccaaaaccaccctgaaagcacagcagaattcaatgattctgactccggcatttcactaaacacaagtcccagtgtggcatcaccagaacactcagtggaatcttccagctatggagacacactacttggcctcagtgattctgaagtggaagagctagatagtgcccctggaagtgtcaaacagaatggtcctaaaacaccagtacattcttctggggatatggtacaacccttgtcaccatctcaggggcagagcactcacgtgcatgatgcccaatgtgagaacacaccagagaaagaattgcctgtaagtcctggtcatcggaaaaccccattcacaaaagacaaacattcaagccgcttggaggctcatctcacaagagatgaacttagggcaaaagctctccatatcccattccctgtagaaaaaatcattaacctccctgttgttgacttcaacgaaatgatgtccaaagagcagttcaatgaagctcaacttgcattaattcgggatatacgtaggaggggtaagaataaagtggctgctcagaattgcagaaaaagaaaactggaaaatatagtagaactagagcaagatttagatcatttgaaagatgaaaaagaaaaattgctcaaagaaaaaggagaaaatgacaaaagccttcacctactgaaaaaacaactcagcaccttatatctcgaagttttcagcatgctacgtgatgaagatggaaaaccttattctcctagtgaatactccctgcagcaaacaagagatggcaatgttttccttgttcccaaaagtaagaagccagatgttaagaaaaactag

SH1：ccctgttgatttagacggtat

| S | AATTGccctgttgatttagacggtatTCAAGAGataccgtctaaatcaacagggTTTTTT |
| --- | --- |
| A | GATCAAAAAAccctgttgatttagacggtatCTCTTGAataccgtctaaatcaacagggC |

SH2：ccggcatttcactaaacacaa

| S | AATTGccggcatttcactaaacacaaTCAAGAGttgtgtttagtgaaatgccggTTTTTT |
| --- | --- |
| A | GATCAAAAAAccggcatttcactaaacacaaCTCTTGAttgtgtttagtgaaatgccggC |

SH3：gcagcaaacaagagatggcaa

| S | AATTGgcagcaaacaagagatggcaaTCAAGAGttgccatctcttgtttgctgcTTTTTT |
| --- | --- |
| A | GATCAAAAAAgcagcaaacaagagatggcaaCTCTTGAttgccatctcttgtttgctgcC |
