# Supplementary material for: Cancer-associated fibroblasts secrete FGF5 to inhibit ferroptosis to decrease cisplatin sensitivity in nasopharyngeal carcinoma through binding to FGFR2
Source: Cell Death Dis. 2024 Apr 18;15(4):279. doi: 10.1038/s41419-024-06671-0 (PMC11026472; doi:10.1038/s41419-024-06671-0)
Supplement: Supplementary file 2 — Original Data File [file 41419_2024_6671_MOESM2_ESM.pdf]

Uncropped western blots:

Fig.1B

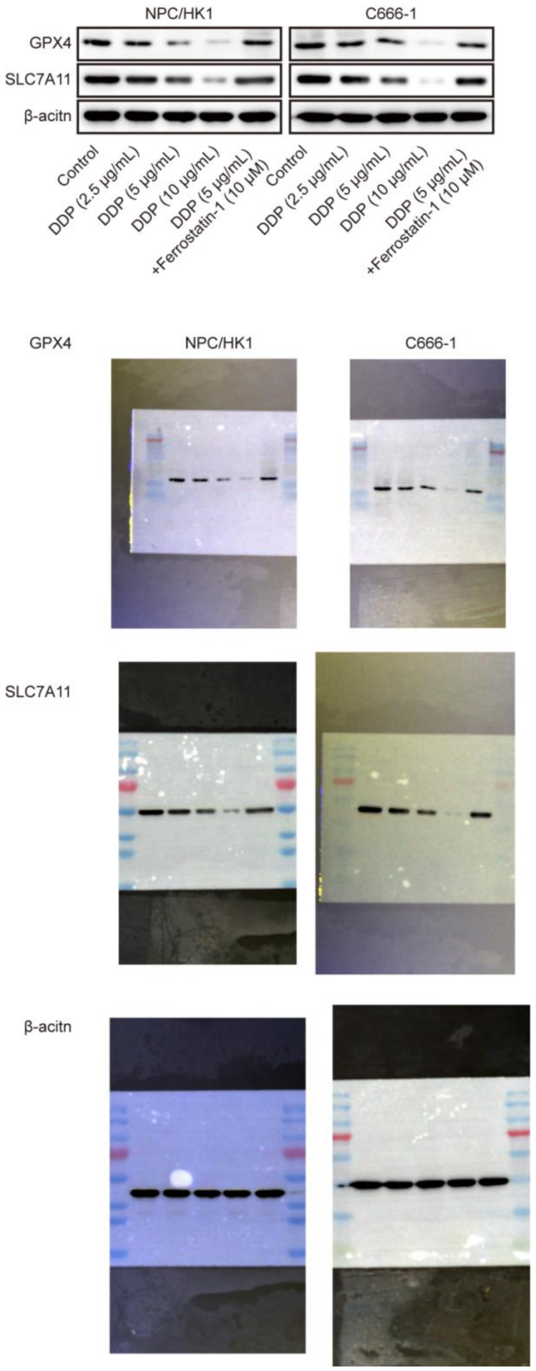

Fig.2E

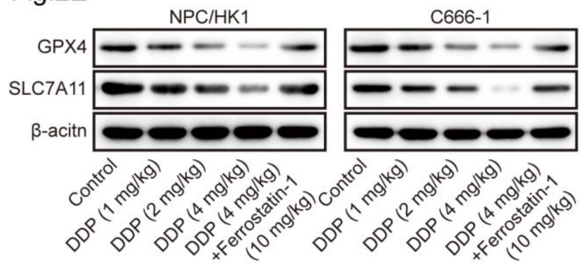

GPX4 NPC/HK1 C666-1

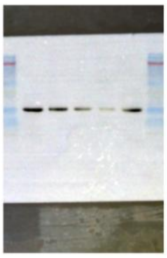

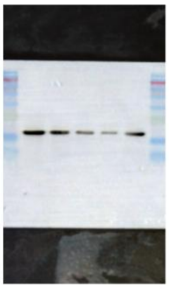

SLC7A11

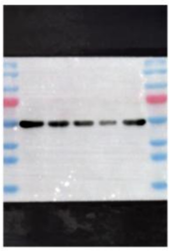

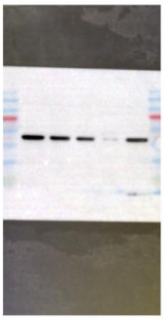

β-acitn

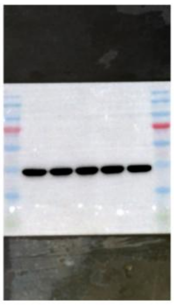

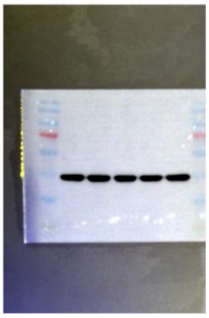

Fig.3B

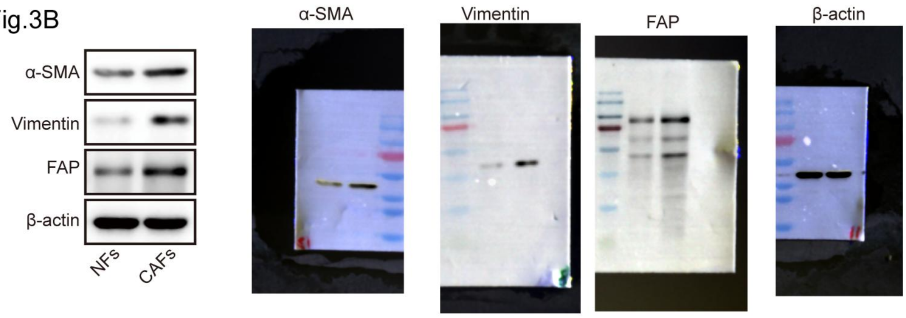

Fig.3C

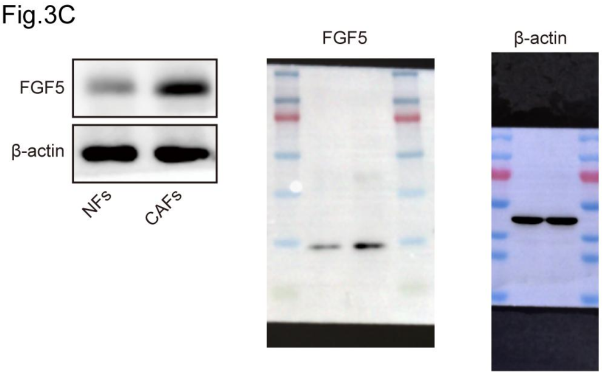

Fig.3F

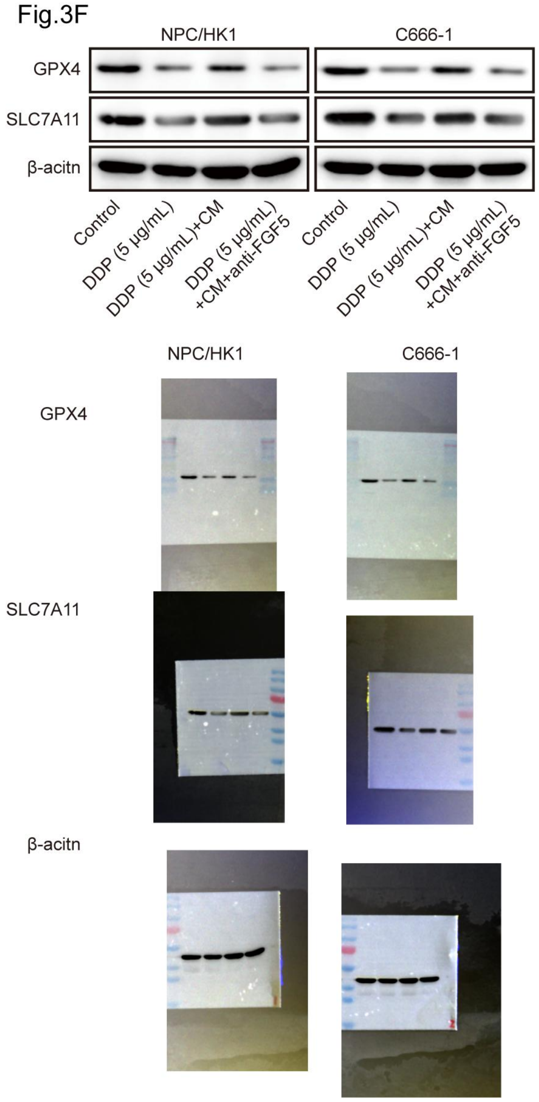

Fig.4B

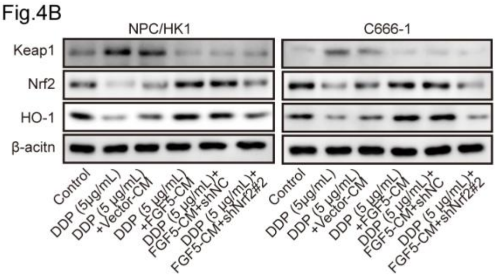
NPC/HK1
C666-1
Keap1
Nrf2
HO-1
β-acitn
Control
DDP (5μg/mL)
DDP (5 μg/mL) +Vector-CM
DDP (5 μg/mL) +FGF5-CM
DDP (5 μg/mL)+ FGF5-CM+shNC
DDP (5 μg/mL)+ FGF5-CM+shNrf2#2

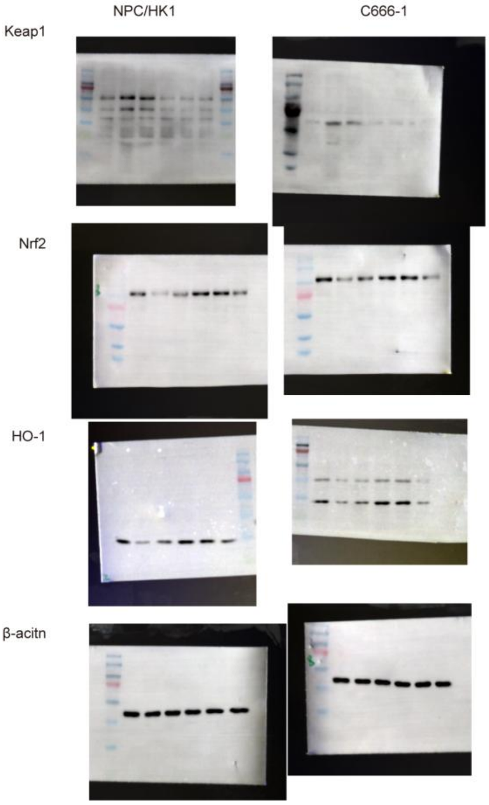
NPC/HK1
C666-1
Keap1
Nrf2
HO-1
β-acitn

Fig.4H

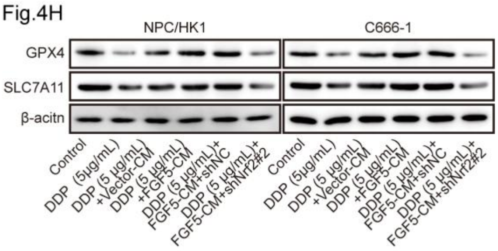
NPC/HK1
C666-1
GPX4
SLC7A11
β-acitn
Control
DDP (5μg/mL)
DDP (5 μg/mL) +Vector-CM
DDP (5 μg/mL) +FGF5-CM
DDP (5 μg/mL)+ FGF5-CM+shNC
DDP (5 μg/mL)+ FGF5-CM+shNrf2#2

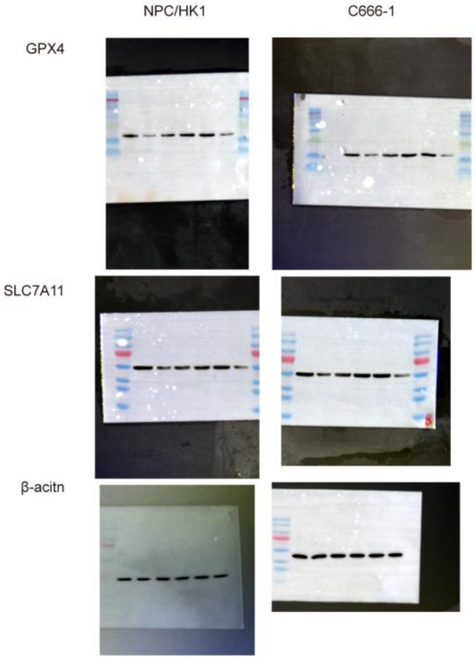
NPC/HK1
C666-1
GPX4
SLC7A11
β-acitn

Fig.5A

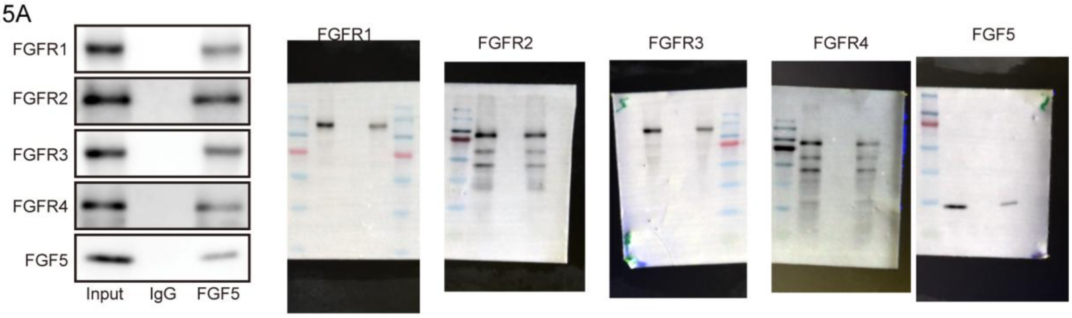
FGFR1
FGFR2
FGFR3
FGFR4
FGF5
Input
IgG
FGF5
FGFR1
FGFR2
FGFR3
FGFR4
FGF5

Fig.5C

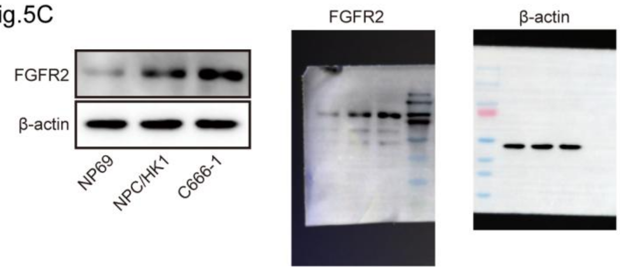
FGFR2
β-actin
NP69
NPC/HK1
C666-1
FGFR2
β-actin

Fig.6A

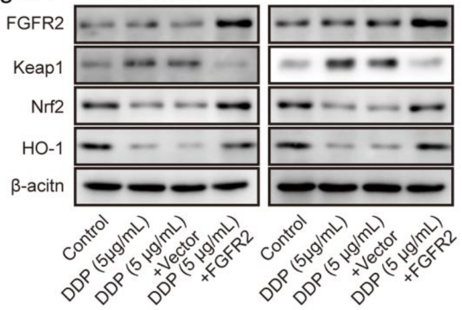
NPC/HK1
C666-1
FGFR2
Keap1
Nrf2
HO-1
β-acitn
Control
DDP (5μg/mL)
DDP (5 μg/mL) +Vector
DDP (5 μg/mL) +FGFR2
Control
DDP (5μg/mL)
DDP (5 μg/mL) +Vector
DDP (5 μg/mL) +FGFR2

Fig.6F

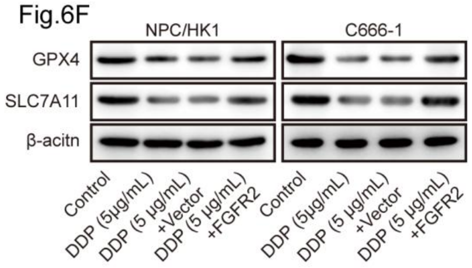
NPC/HK1
C666-1
GPX4
SLC7A11
β-acitn
Control
DDP (5μg/mL)
DDP (5 μg/mL) +Vector
DDP (5 μg/mL) +FGFR2
Control
DDP (5μg/mL)
DDP (5 μg/mL) +Vector
DDP (5 μg/mL) +FGFR2

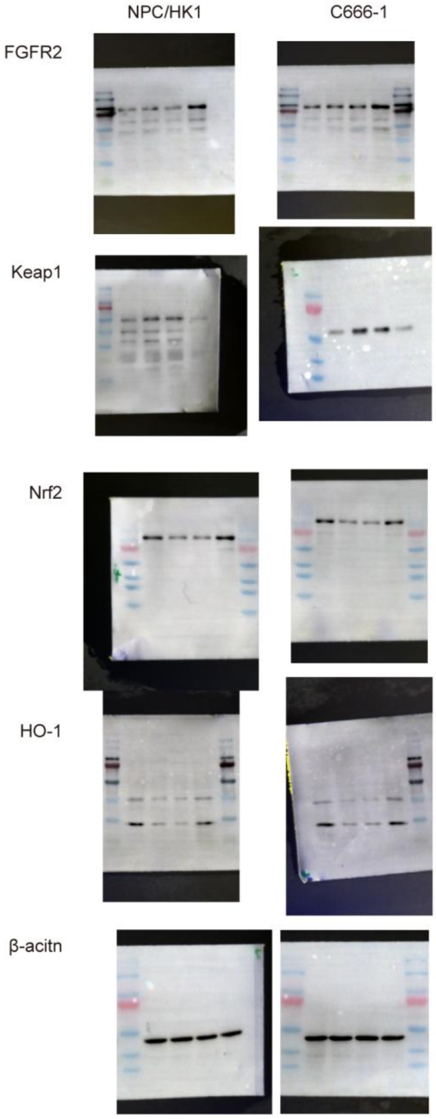
NPC/HK1
C666-1
FGFR2
Keap1
Nrf2
HO-1
β-acitn

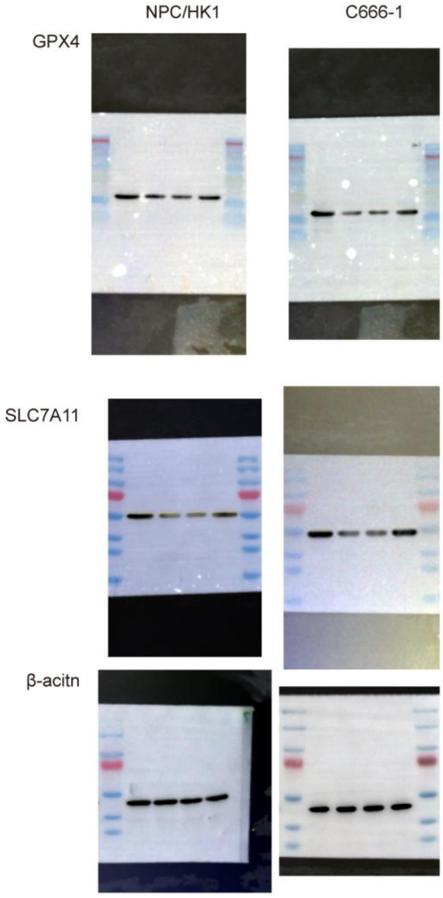
NPC/HK1
C666-1
GPX4
SLC7A11
β-acitn

Fig.7B

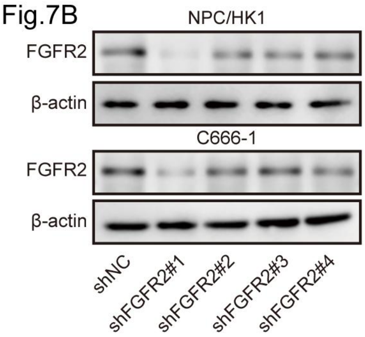
NPC/HK1
FGFR2
β-actin
C666-1
FGFR2
β-actin
shNC
shFGFR2#1
shFGFR2#2
shFGFR2#3
shFGFR2#4

Fig.7C

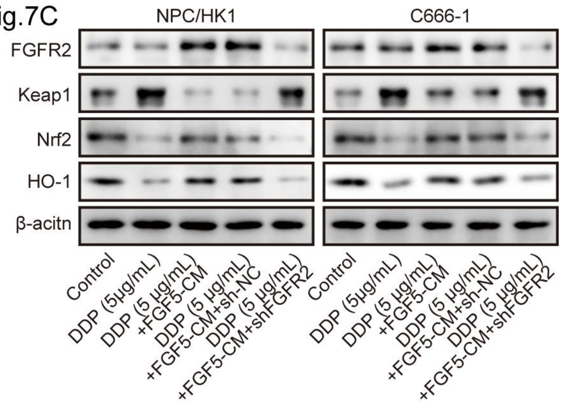
NPC/HK1
C666-1
FGFR2
Keap1
Nrf2
HO-1
β-acitn
Control
DDP (5μg/mL)
DDP (5 μg/mL) +FGF5-CM
DDP (5 μg/mL) +FGF5-CM+sh-NC
DDP (5 μg/mL) +FGF5-CM+shFGFR2
Control
DDP (5μg/mL)
DDP (5 μg/mL) +FGF5-CM
DDP (5 μg/mL) +FGF5-CM+sh-NC
DDP (5 μg/mL) +FGF5-CM+shFGFR2

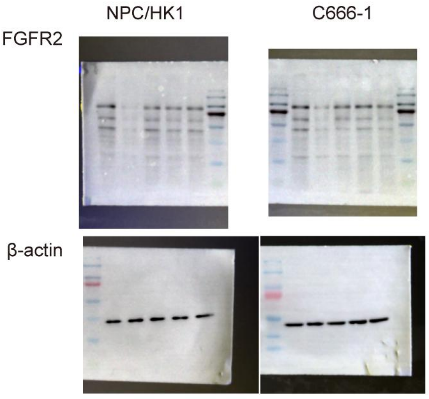
NPC/HK1
C666-1
FGFR2
β-actin

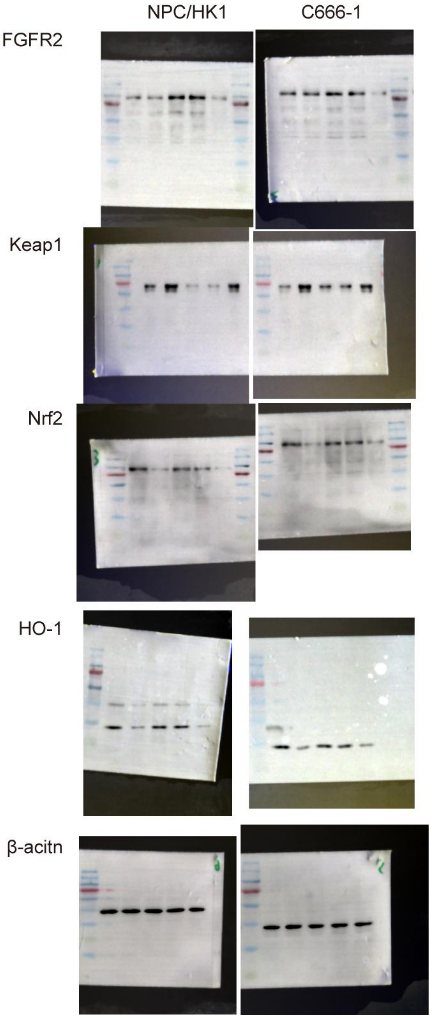
NPC/HK1
C666-1
FGFR2
Keap1
Nrf2
HO-1
β-acitn

Fig.8H

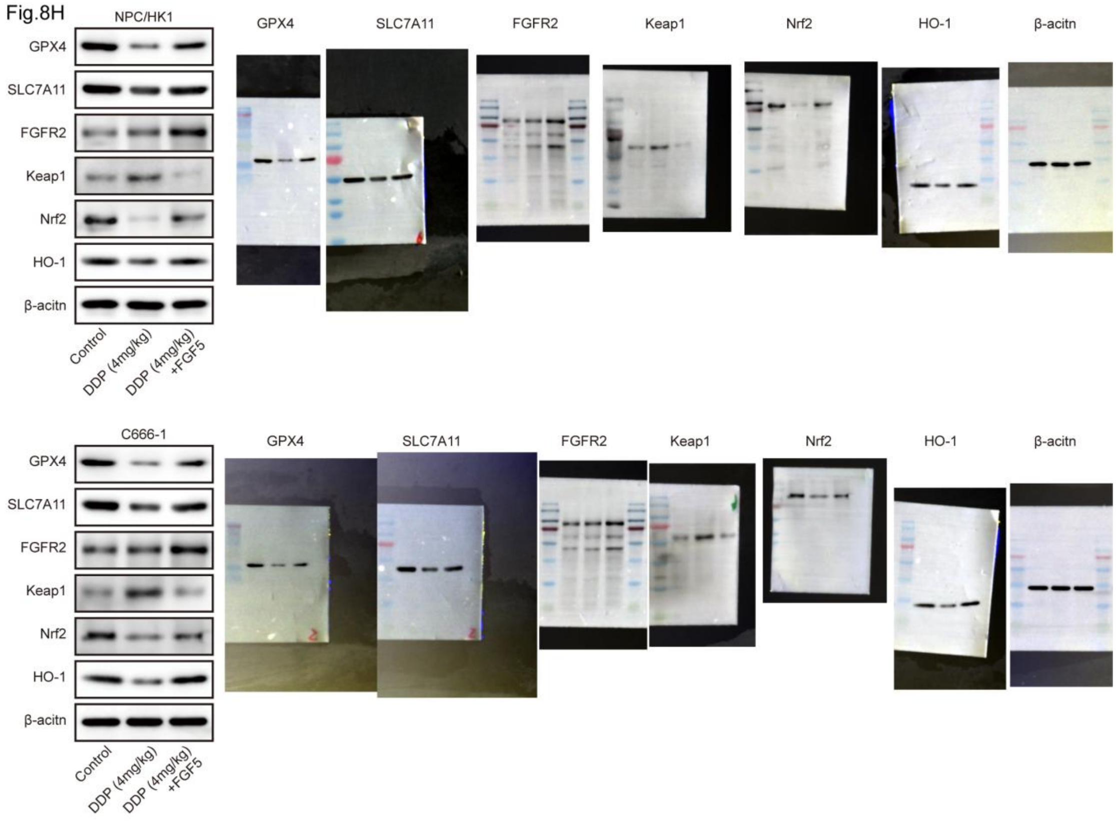
NPC/HK1
GPX4
SLC7A11
FGFR2
Keap1
Nrf2
HO-1
β-acitn
Control
DDP (4mg/kg)
DDP (4mg/kg) +FGF5
GPX4
SLC7A11
FGFR2
Keap1
Nrf2
HO-1
β-acitn
C666-1
GPX4
SLC7A11
FGFR2
Keap1
Nrf2
HO-1
β-acitn
Control
DDP (4mg/kg)
DDP (4mg/kg) +FGF5
GPX4
SLC7A11
FGFR2
Keap1
Nrf2
HO-1
β-acitn

Fig.S1A

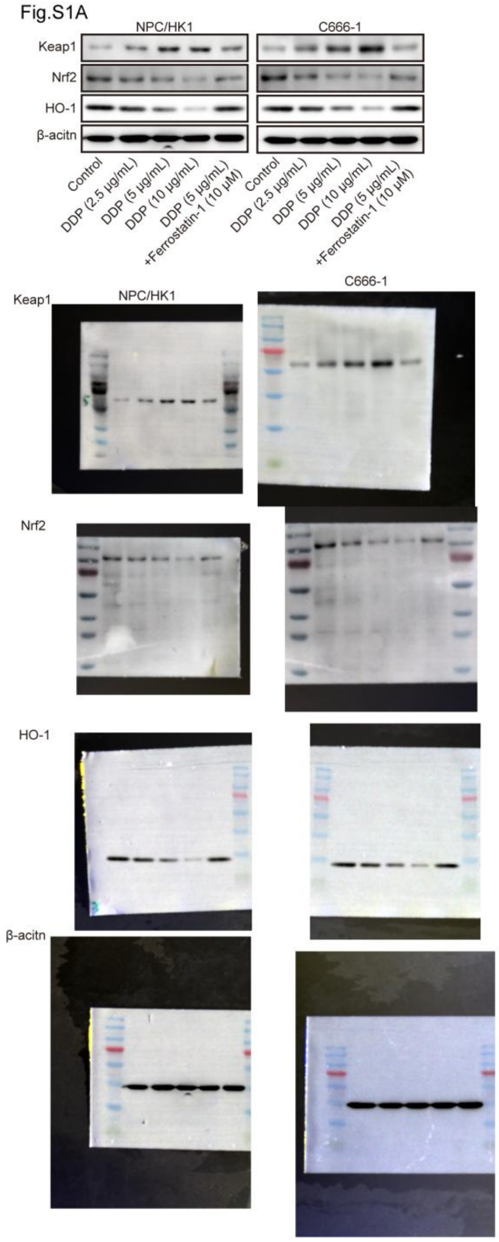

Fig.S1E

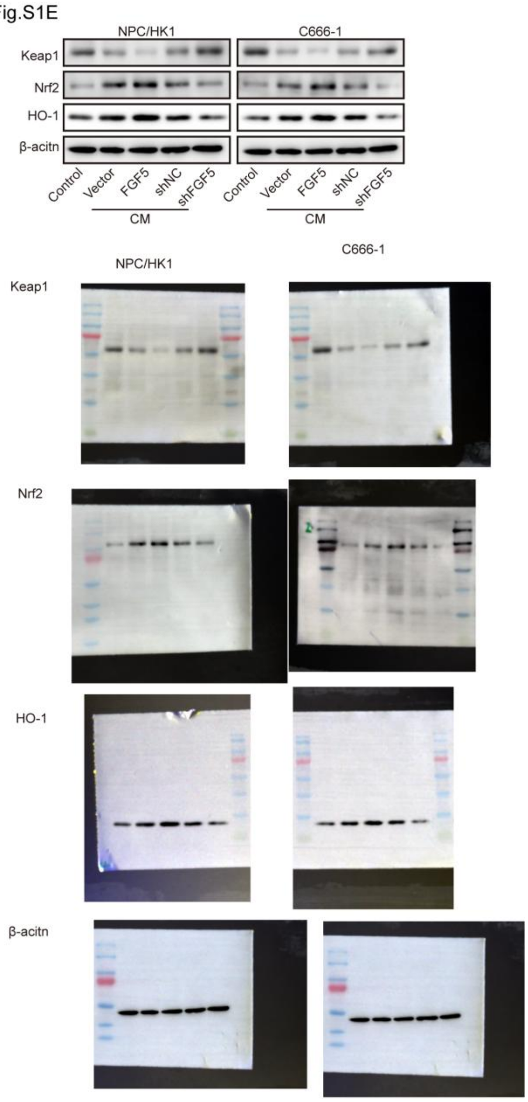

Fig.S2A

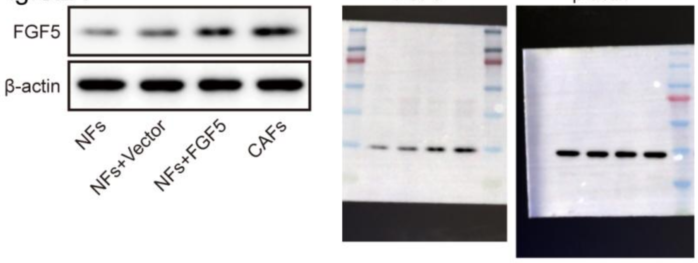

Fig.S2H

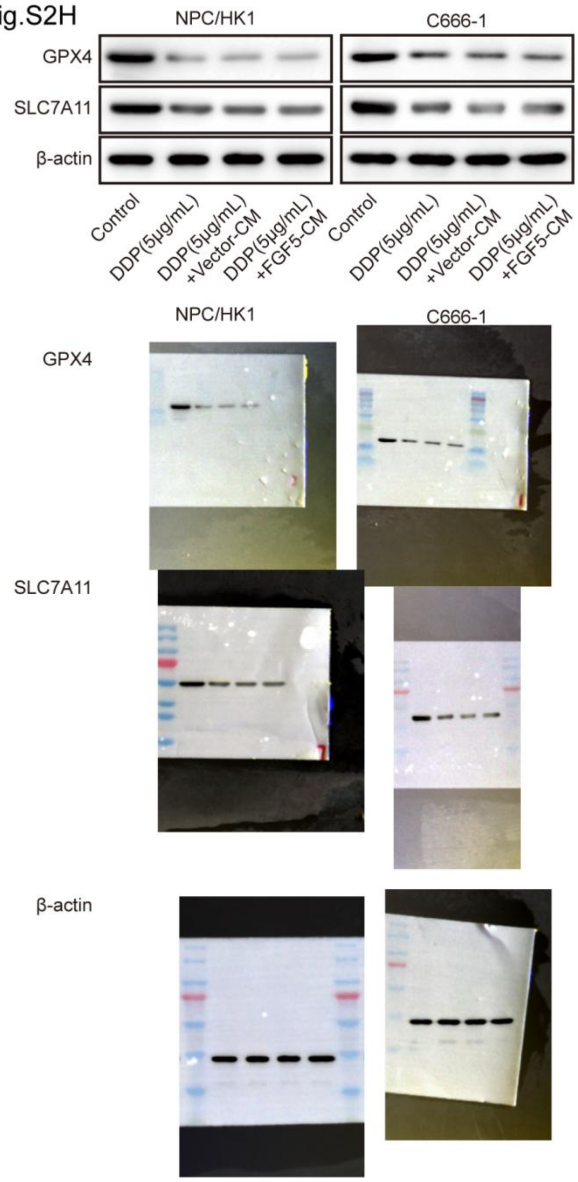

Sequences of shRNAs:

Homo sapiens fibroblast growth factor 5 (FGF5), transcript variant 1, mRNA

NCBI Reference Sequence: NM_004464.4

atgagcttgtccttcctcctcctcttcttcagccacctgatcctcagcgcctgggctcacggggagaagcgtctcgcccccaaagggcaacccggacccgctgccac
tgataggaaccctagaggctccagcagcagacagagcagcagtagcgctatgtcttcctcttctgcctcctcctcccccgcagcttctctgggcagccaaggaagtggc
ttggagcagagcagtttccagtggagcccctcggggcgccggaccggcagcctctactgcagagtgggcatcggtttccatctgcagatctacccggatggcaaagtc
aatggatcccacgaagccaatatgttaagtgttttggaaatatttgctgtgtctcaggggattgtaggaatacgaggagttttcagcaacaaatttttagcgatgtcaaa
aaaaggaaaactccatgcaagtgccaagttcacagatgactgcaagttcagggagcgttttcaagaaaatagctataatacctatgcctcagcaatacatagaactg
aaaaaacagggcgggagtggtatgtggccctgaataaaagaggaaaagccaaacgagggtgcagcccccgggttaaacccagcatatctctacccattttctgcc
aagattcaagcagtcggagcagccagaactttctttcacggttactgttcctgaaaagaaaaagccacctagccctatcaagccaaagattcccctttctgcacctcgg
aaaaataccaactcagtgaaatacagactcaagtttcgctttggataa

SH1：cccagcatatctctacccatt

S	AATTGcccagcatatctctacccattTCAAGAGaatgggtagagatatgctgggTTTTTT
A	GATCAAAAAAcccagcatatctctacccattCTCTTGAaatgggtagagatatgctgggC

SH2：cctatgcctcagcaatacata

S	AATTGcctatgcctcagcaatacataTCAAGAGtatgtattgctgaggcataggTTTTTT
A	GATCAAAAAAcctatgcctcagcaatacataCTCTTGAtatgtattgctgaggcataggC

SH3：gaagccaatatgttaagtgtt

S	AATTGgaagccaatatgttaagtgttTCAAGAGaacacttaacatattggcttcTTTTTT
A	GATCAAAAAAgaagccaatatgttaagtgttCTCTTGAaacacttaacatattggcttcC

SH4：ccagaactttctttcacggtt

S	AATTGccagaactttctttcacggttTCAAGAGaaccgtgaaagaaagttctggTTTTTT
A	GATCAAAAAAccagaactttctttcacggttCTCTTGAaaccgtgaaagaaagttctggC

Homo sapiens fibroblast growth factor receptor 2 (FGFR2), transcript variant 1, mRNA

NCBI Reference Sequence: NM_000141.5

atggtcagctggggtcgtttcatctgcctggtcgtggtcaccatggcaaccttgtccctggcccggccctccttcagtttagttgaggataccacattagagccagaaga
gccaccaaccaaataccaaatctctcaaccagaagtgtacgtggctgcgccaggggagtcgctagaggtgcgctgcctgttgaaagatgccgccgtgatcagttgga
ctaaggatggggtgcacttggggcccaacaataggacagtgcttattggggagtacttgcagataaagggcgccacgcctagagactccggcctctatgcttgtactg
ccagtaggactgtagacagtgaaacttggtacttcatggtgaatgtcacagatgccatctcatccggagatgatgaggatgacaccgatggtgcggaagattttgtcag
tgagaacagtaacaacaagagagcaccatactggaccaacacagaaaagatggaaaagcggctccatgctgtgcctgcggccaacactgtcaagtttcgctgccca
gccgggggggaacccaatgccaaccatgcggtggctgaaaaacgggaaggagtttaagcaggagcatcgcattggaggctacaaggtacgaaaccagcactggag
cctcattatggaaagtgtggtcccatctgacaagggaaattatacctgtgtagtggagaatgaatacgggtccatcaatcacacgtaccacctggatgttgtggagcga
tcgcctcaccggcccatcctccaagccggactgccggcaaatgcctccacagtggtcggaggagacgtagagtttgtctgcaaggtttacagtgatgcccagccccac
atccagtggatcaagcacgtggaaaagaacggcagtaaatacgggcccgacgggctgccctacctcaaggttctcaaggccgccggtgttaacaccacggacaaag
agattgaggttctctatattcggaatgtaacttttgaggacgctggggaatatacgtgcttggcgggtaattctattgggatatcctttcactctgcatggttgacagttct
gccagcgcctggaagagaaaaggagattacagcttccccagactacctggagatagccatttactgcataggggtcttcttaatcgcctgtatggtggtaacagtcatc

ctgtgccgaatgaagaacacgaccaagaagccagacttcagcagccagccggctgtgcacaagctgaccaaacgtatccccctgcggagacaggtaacagtttcgg
ctgagtccagctcctccatgaactccaacaccccgctggtgaggataacaacacgcctctcttcaacggcagacacccccatgctggcaggggtctccgagtatgaac
ttccagaggacccaaaatgggagtttccaagagataagctgacactgggcaagcccctgggagaaggttgctttgggcaagtggtcatggcggaagcagtgggaatt
gacaaagacaagcccaaggaggcggtcaccgtggccgtgaagatgttgaaagatgatgccacagagaaagacctttctgatctggtgtcagagatggagatgatga
agatgattgggaaacacaagaatatcataaatcttcttggagcctgcacacaggatgggcctctctatgtcatagttgagtatgcctctaaaggcaacctccgagaata
cctccgagcccggaggccacccgggatggagtactcctatgacattaaccgtgttcctgaggagcagatgaccttcaaggacttggtgtcatgcacctaccagctggcc
agaggcatggagtacttggcttcccaaaaatgtattcatcgagatttagcagccagaaatgttttggtaacagaaaacaatgtgatgaaaatagcagactttggactcg
ccagagatatcaacaatatagactattacaaaaagaccaccaatgggcggcttccagtcaagtggatggctccagaagccctgtttgatagagtatacactcatcaga
gtgatgtctggtccttcggggtgttaatgtgggagatcttcactttagggggctcgccctacccagggattcccgtggaggaactttttaagctgctgaaggaaggacac
agaatggataagccagccaactgcaccaacgaactgtacatgatgatgagggactgttggcatgcagtgccctcccagagaccaacgttcaagcagttggtagaaga
cttggatcgaattctcactctcacaaccaatgaggaatacttggacctcagccaacctctcgaacagtattcacctagttaccctgacacaagaagttcttgttcttcagg
agatgattctgtttttctccagaccccatgccttacgaaccatgccttcctcagtatccacacataaacggcagtgttaaaacatga

SH1：agccctgtttgatagagtata

S	AATTGagccctgtttgatagagtataTCAAGAGtatactctatcaaacagggctTTTTTT
A	GATCAAAAAAagccctgtttgatagagtataCTCTTGAtatactctatcaaacagggctC

SH2：ttagttgaggataccacatta

S	AATTGttagttgaggataccacattaTCAAGAGtaatgtggtatcctcaactaaTTTTTT
A	GATCAAAAAAttagttgaggataccacattaCTCTTGAtaatgtggtatcctcaactaaC

SH3：gccaccaaccaaataccaaat

S	AATTGgccaccaaccaaataccaaatTCAAGAGatttggtatttggttggtggcTTTTTT
A	GATCAAAAAAgccaccaaccaaataccaaatCTCTTGAatttggtatttggttggtggcC

SH4：cagtgaaacttggtacttcat

S	AATTGcagtgaaacttggtacttcatTCAAGAGatgaagtaccaagtttcactgTTTTTT
A	GATCAAAAAAcagtgaaacttggtacttcatCTCTTGAatgaagtaccaagtttcactgC

Homo sapiens NFE2 like bZIP transcription factor 2 (NFE2L2), transcript variant 1, mRNA

NCBI Reference Sequence: NM_006164.5

atgatggacttggagctgccgccgccgggactcccgtcccagcaggacatggatttgattgacatactttggaggcaagatatagatcttggagtaagtcgagaagtat
ttgacttcagtcagcgacggaaagagtatgagctggaaaaacagaaaaaacttgaaaaggaaagacaagaacaactccaaaaggagcaagagaaagcctttttcg
ctcagttacaactagatgaagagacaggtgaatttctcccaattcagccagcccagcacatccagtcagaaaccagtggatctgccaactactcccaggttgcccacat
tcccaaatcagatgctttgtactttgatgactgcatgcagcttttggcgcagacattcccgtttgtagatgacaatgaggtttcttcggctacgtttcagtcacttgttcctga
tattcccggtcacatcgagagcccagtcttcattgctactaatcaggctcagtcacctgaaacttctgttgctcaggtagcccctgttgatttagacggtatgcaacagga
cattgagcaagtttgggaggagctattatccattcctgagttacagtgtcttaatattgaaaatgacaagctggttgagactaccatggttccaagtccagaagccaaac
tgacagaagttgacaattatcatttttactcatctataccctcaatggaaaaagaagtaggtaactgtagtccacattttcttaatgcttttgaggattccttcagcagcat
cctctccacagaagaccccaaccagttgacagtgaactcattaaattcagatgccacagtcaacacagattttggtgatgaattttattctgctttcatagctgagcccag
tatcagcaacagcatgccctcacctgctactttaagccattcactctctgaacttctaaatgggcccattgatgtttctgatctatcactttgcaaagctttcaaccaaaac
caccctgaaagcacagcagaattcaatgattctgactccggcatttcactaaacacaagtcccagtgtggcatcaccagaacactcagtggaatcttccagctatggag
acacactacttggcctcagtgattctgaagtggaagagctagatagtgcccctggaagtgtcaaacagaatggtcctaaaacaccagtacattcttctggggatatggt

acaacccttgtcaccatctcaggggcagagcactcacgtgcatgatgcccaatgtgagaacacaccagagaaagaattgcctgtaagtcctggtcatcggaaaaccc
cattcacaaaagacaaacattcaagccgcttggaggctcatctcacaagagatgaacttagggcaaaagctctccatatcccattccctgtagaaaaaatcattaacc
tccctgttgttgacttcaacgaaatgatgtccaaagagcagttcaatgaagctcaacttgcattaattcgggatatacgtaggaggggtaagaataaagtggctgctca
gaattgcagaaaaagaaaactggaaaatatagtagaactagagcaagatttagatcatttgaaagatgaaaaagaaaaattgctcaaagaaaaaggagaaaatga
caaaagccttcacctactgaaaaaacaactcagcaccttatatctcgaagttttcagcatgctacgtgatgaagatggaaaaccttattctcctagtgaatactccctgc
agcaaacaagagatggcaatgttttccttgttcccaaaagtaagaagccagatgttaagaaaaactag

SH1： ccctgttgatttagacggtat

S	AATTGccctgttgatttagacggtatTCAAGAGataccgtctaaatcaacagggTTTTTT
A	GATCAAAAAAccctgttgatttagacggtatCTCTTGAataccgtctaaatcaacagggC

SH2： ccggcatttcactaaacacaa

S	AATTGccggcatttcactaaacacaaTCAAGAGttgtgtttagtgaaatgccggTTTTTT
A	GATCAAAAAAccggcatttcactaaacacaaCTCTTGAttgtgtttagtgaaatgccggC

SH3： gcagcaaacaagagatggcaa

S	AATTGgcagcaaacaagagatggcaaTCAAGAGttgccatctcttgtttgctgcTTTTTT
A	GATCAAAAAAgcagcaaacaagagatggcaaCTCTTGAttgccatctcttgtttgctgcC
